# The complete genome sequence of the nitrile biocatalyst *Rhodocccus rhodochrous* ATCC BAA-870

**DOI:** 10.1186/s12864-019-6405-7

**Published:** 2020-01-02

**Authors:** Joni Frederick, Fritha Hennessy, Uli Horn, Pilar de la Torre Cortés, Marcel van den Broek, Ulrich Strych, Richard Willson, Charles A. Hefer, Jean-Marc G. Daran, Trevor Sewell, Linda G. Otten, Dean Brady

**Affiliations:** 1 Protein Technologies, CSIR Biosciences, Meiring Naude Road, Brummeria, Pretoria, South Africa; 2 Electron Microscope Unit, University of Cape Town, Rondebosch, 7701 South Africa; 3 Present Address: LadHyx, UMR CNRS 7646, École Polytechnique, 91128 Palaiseau, France; 4 Meraka, CSIR, Meiring Naude Road, Brummeria, 0091 South Africa; 5 Industrial Microbiology, Department of Biotechnology, Delft University of Technology, Van der Maasweg 9, 2629 HZ Delft, The Netherlands; 6 Biology and Biochemistry, University of Houston, 4800 Calhoun Road, Houston, TX 77204 USA; 7 Present Address: Department of Pediatrics, Section of Tropical Medicine, Baylor College of Medicine, 1102 Bates Avenue, Houston, TX 77030 USA; 8 Chemical and Biomolecular Engineering, University of Houston, 4800 Calhoun Road, Houston, TX 77204 USA; 9 Bioinformatics and Computational Biology Unit, Department of Biochemistry, Genetics and Microbiology, University of Pretoria, Pretoria, 0002 South Africa; 10 Present Address: AgResearch Limited, Lincoln Research Centre, Private Bag 4749, Christchurch, 8140 New Zealand; 11 Biocatalysis, Department of Biotechnology, Delft University of Technology, Van der Maasweg 9, 2629 HZ Delft, The Netherlands; 12 Molecular Sciences Institute, School of Chemistry, University of the Witwatersrand, PO, Wits, 2050 South Africa

## Abstract

**Background:**

Rhodococci are industrially important soil-dwelling Gram-positive bacteria that are well known for both nitrile hydrolysis and oxidative metabolism of aromatics. *Rhodococcus rhodochrous* ATCC BAA-870 is capable of metabolising a wide range of aliphatic and aromatic nitriles and amides. The genome of the organism was sequenced and analysed in order to better understand this whole cell biocatalyst.

**Results:**

The genome of *R. rhodochrous* ATCC BAA-870 is the first *Rhodococcus* genome fully sequenced using Nanopore sequencing. The circular genome contains 5.9 megabase pairs (Mbp) and includes a 0.53 Mbp linear plasmid, that together encode 7548 predicted protein sequences according to BASys annotation, and 5535 predicted protein sequences according to RAST annotation. The genome contains numerous oxidoreductases, 15 identified antibiotic and secondary metabolite gene clusters, several terpene and nonribosomal peptide synthetase clusters, as well as 6 putative clusters of unknown type. The 0.53 Mbp plasmid encodes 677 predicted genes and contains the nitrile converting gene cluster, including a nitrilase, a low molecular weight nitrile hydratase, and an enantioselective amidase.

Although there are fewer biotechnologically relevant enzymes compared to those found in rhodococci with larger genomes, such as the well-known *Rhodococcus jostii* RHA1, the abundance of transporters in combination with the myriad of enzymes found in strain BAA-870 might make it more suitable for use in industrially relevant processes than other rhodococci.

**Conclusions:**

The sequence and comprehensive description of the *R. rhodochrous* ATCC BAA-870 genome will facilitate the additional exploitation of rhodococci for biotechnological applications, as well as enable further characterisation of this model organism. The genome encodes a wide range of enzymes, many with unknown substrate specificities supporting potential applications in biotechnology, including nitrilases, nitrile hydratase, monooxygenases, cytochrome P450s, reductases, proteases, lipases, and transaminases.

## Background

*Rhodococcus* is arguably the most industrially important actinomycetes genus [1] owing to its wide-ranging applications as a biocatalyst used in the synthesis of pharmaceuticals [2], in bioactive steroid production [3], fossil fuel desulphurization [4], and the production of kilotons of commodity chemicals [5]. Rhodococci have been shown to have a variety of important enzyme activities in the field of biodegradation (for reviews see [6, 7]). These activities could also be harnessed for synthesis of various industrially relevant compounds [8]. One of the most interesting qualities of rhodococci that make them suitable for use in industrial biotechnology is their outer cell wall [9]. It is highly hydrophobic through a high percentage of mycolic acid, which promotes uptake of hydrophobic compounds. Furthermore, upon contact with organic solvents, the cell wall composition changes, becoming more resistant to many solvents and more stable under industrially relevant conditions like high substrate concentration and relatively high concentrations of both water-miscible and -immiscible solvents. This results in a longer lifetime of the whole cell biocatalyst and subsequent higher productivity.

Rhodococcal species isolated from soil are known to have diverse catabolic activities, and their genomes hold the key to survival in complex chemical environments [10]. The first full *Rhodococcus* genome sequenced was that of *Rhodococcus jostii* RHA1 (NCBI database: NC_008268.1) in 2006 [10]. *R. jostii* RHA1 was isolated in Japan from soil contaminated with the toxic insecticide lindane (γ-hexachlorocyclohexane) [11] and was found to degrade a range of polychlorinated biphenyls (PCBs) [12]. Its full genome is 9.7 Mbp, inclusive of the 7.8 Mbp chromosome and 3 plasmids (pRHL1, 2 and 3). Since then, many additional rhodococci have been sequenced by various groups and consortia (Additional file 1: Table S1). One sequencing effort to improve prokaryotic systematics has been implemented by the University of Northumbria, which showed that full genome sequencing provides a robust basis for the classification and identification of rhodococci that have agricultural, industrial and medical/veterinary significance [13].

A few rhodococcal genomes have been more elaborately described (Table 1), including *R. erythropolis* PR4 (NC_012490.1) [18] which degrades long alkanes [19]. Multiple monooxygenases and fatty acid β-oxidation pathway genes were found on the *R. erythropolis* PR4 genome and several plasmids, making this bacterium a perfect candidate for bioremediation of hydrocarbon-contaminated sites and biodegradation of animal fats and vegetable oils. The related *R. rhodochrous* ATCC 17895 (NZ_ASJJ01000002) [20] also has many mono- and dioxygenases, as well as interesting hydration activities which could be of value for the organic chemist. The oleaginous bacterium *R. opacus* PD630 is a very appealing organism for the production of biofuels and was sequenced by two separate groups. Holder et al. used enrichment culturing of *R. opacus* PD630 to analyse the lipid biosynthesis of the organism, and the ~ 300 or so genes involved in oleaginous metabolism [16]. This sequence is being used in comparative studies for biofuel development. The draft sequence of the *R. opacus* PD630 genome was only recently released (NZ_AGVD01000000) and appears to be 9.15 Mbp, just slightly smaller than that of *R. jostii* RHA1. The full sequence of the same strain was also deposited in 2012 by Chen et al. (NZ_CP003949) [15], who focused their research on the lipid droplets of this strain. Twenty strains of *R. fascians* were sequenced to understand the pathogenicity of this species for plants [21], which also resulted in the realisation that sequencing provides additional means to traditional ways of determining speciation in the very diverse genus of *Rhodococcus* [22]. The clinically important pathogenic strain *R. hoagii* 103S (formerly known as *R. equi* 103S) was also fully sequenced in order to understand its biology and virulence evolution (NC_014659.1) [17]. In this and other pathogenic *R. hoagii* strains, virulence genes are usually located on plasmids, which was well described for several strains including ATCC 33701 and 103 [23], strain PAM1593 [24] and 96 strains isolated from Normandy (France) [25]. As many important traits are often located on (easily transferable) plasmids, numerous rhodococcal plasmid sequences have been submitted to the NCBI (Additional file 1: Table S2). More elaborate research has been published on the virulence plasmid pFiD188 from *R. fascians* D188 [26], pB264, a cryptic plasmid from *Rhodococcus* sp. B264–1 [27], pNC500 from *R. rhodochrous* B-276 [28], and several plasmids from *R. opacus* B4 [29] and PD630 [15]. *R. erythropolis* harbours many plasmids besides the three from strain PR4, including pRE8424 from strain DSM8424 [30], pFAJ2600 from NI86/21 [31] and pBD2 from strain BD2 [32]. All these sequences have highlighted the adaptability of rhodococci and explain the broad habitat of this genus.

**Table 1:** Fully sequenced^a^ and well described *Rhodococcus* species ranked by completion date

Organism	Date Completed^b^	Group	Reference	Chromosome (Mbp)	Plasmid (Mbp)	Total Size, Mbp	G + C %	Protein coding genes
*R. rhodochrous* ATCC BAA-870	2018	This study	This paper	5.37	0.53	5.9	65	7548^e^
*R. erythropolis* R138	19-03-2013	Centre National de la Recherche Scientifique, Institut des Sciences du Vegetal, France	NZ_CP007255 [14]	6.2	477,915; 91,729	6.8	62	6130
*R. opacus* PD630^c^	26-11-2012	National Laboratory of Macromolecules, Chinese Academy of Sciences, Beijing	NZ_CP003949 [15]	8.38	9 plasmids	9.17	67	8947
*R. opacus* PD630^c^	10-11-2011	Massachusetts Institute of Technology and The Broad Institute	GCF_000234335 [16]	–	–	9.27	67	7910
*R. hoagii* 103S^d^	21-10-2009	IREC (International *Rhodococcus equi* Genome Consortium)	NC_014659 [17]	5.04	None determined	5.04	69	4540
*R. jostii* RHA1	24-07-2006	Genome British Columbia, Vancouver	NC_008268 [10]	7.8	1,123,075; 442,536; 332,361	9.7	67	8690
*R. erythropolis* PR4	31-03-2005	Sequencing Center: National Institute of Technology and Evaluation, Japan	NC_012490 [18]	6.5	271,577; 104,014; 3637	6.9	62	6321

^a^All sequences are completed and fully assembled, except GCF_000234335, which consists of 282 contigs

^b^Date completed refers to genome sequence completion/submission to database; plasmids may have been completed at another time. Total genome size comprises the chromosome and the plasmid sequence. Genome information of strains other than BAA-870 is obtained from the NCBI database

^c^Two separate references, therefore 2 entries

^d^*R. equi* is renamed to *R. hoagii*

^e^Based on BASys annotation

The versatile nitrile-degrading bacterium, *R. rhodochrous* ATCC BAA-870 [33], was isolated through enrichment culturing of soil samples from South Africa on nitrile nitrogen sources. *R. rhodochrous* ATCC BAA-870 possesses nitrile-hydrolysing activity capable of metabolising a wide range of aliphatic and aromatic nitriles and amides through the activity of nitrilase, nitrile hydratase and amidase [33–36]. These enzymes can also perform enantioselective hydrolysis of nitrile compounds selected from classes of chemicals used in pharmaceutical intermediates, such as β-adrenergic blocking agents, antitumor agents, antifungal antibiotics and antidiabetic drugs. Interestingly, the nitrile hydratase-amidase system can enantioselectively hydrolyse some compounds, while the nitrilase hydrolyses the opposite enantiomer of similar nitriles [37]. Biocatalytic nitrile hydrolysis affords valuable applications in industry, including production of solvents, extractants, pharmaceuticals, drug intermediates, and pesticides [38–41]. Herein, we describe the sequencing and annotation of *R. rhodochrous* ATCC BAA-870, identifying the genes associated with nitrile hydrolysis as well as other genes for potential biocatalytic applications. The extensive description of this genome and the comparison to other sequenced rhodococci will add to the knowledge of the *Rhodococcus* phylogeny and its industrial capacity.

## Results

### Genome preparation, sequencing and assembly

The genome of *R. rhodochrous* ATCC BAA-870 was originally sequenced in 2009 by Solexa Illumina with sequence reads of average length 36 bps, resulting in a coverage of 74%, with an apparent raw coverage depth of 36x. An initial assembly of this 36-cycle, single-ended Illumina library, together with a mate-pair library, yielded a 6 Mbp genome of 257 scaffolds. A more recently performed paired-end Illumina library combined with the mate-pair library reduced this to only 6 scaffolds (5.88 Mbp). Even after several rounds of linking the mate-pair reads, we were still left with 3 separate contiguous sequences (contigs). The constraint was caused by the existence of repeats in the genome of which one was a 5.2 kb contig that, based on sequence coverage, must exist in four copies, containing 16S-like genes. Applying third generation sequencing (Oxford Nanopore Technology) enabled the full assembly of the genome, while the second generation (Illumina) reads provided the necessary proof-reading. This resulted in a total genome size of 5.9 Mbp, consisting of a 5.37 Mbp circular chromosome and a 0.53 Mbp linear plasmid. The presence of the plasmid was confirmed by performing Pulse Field Gel Electrophoresis using non-digested DNA [42]. The complete genome sequence of *R. rhodochrous* ATCC BAA-870 is deposited at NCBI GenBank, with Bioproject accession number PRJNA487734, and Biosample accession number SAMN09909133.

### Taxonomy and lineage of *R. rhodochrous* ATCC BAA-870

**Fig. 1:**
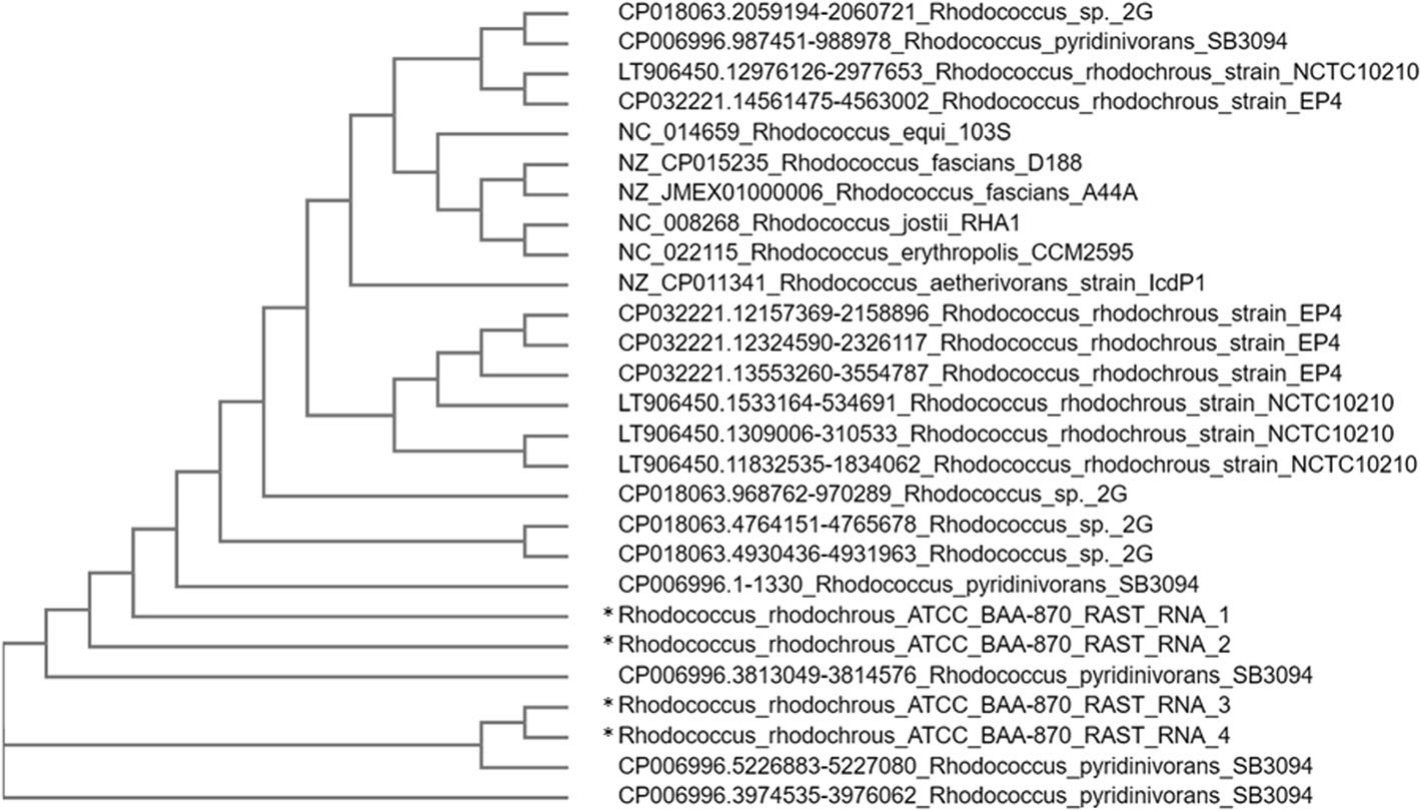
Phylogenetic tree created using rhodococcal 16S rRNA ClustalW sequence alignments. Neighbour joining, phylogenetic cladogram created using Phylogeny in ClustalW, and ClustalO multiple sequence alignment of *R. rhodochrous* ATCC BAA-870 16S rRNA genes and other closely matched genes from rhodococcal species. *R. rhodochrous* ATCC BAA-870 contains four copies of the 16S rRNA gene (labelled RNA_1 to RNA_4) and are indicated with an asterisk. For clarity, only closely matched BLAST results with greater than 95.5% sequence identity and those with complete 16S rRNA gene sequences, or from complete genomes, are considered. Additionally, 16S rRNA gene sequences (obtained from the NCBI gene database) from *R. jostii* RHA1, *R. fascians* A44A and D188, *R. equi* 103S, *R. erythropolis* CCM2595, and *R. aetherivorans* strain IcdP1 are included for comparison. Strain names are preceded by their NCBI accession number, as well as sequence position if there are multiple copies of the 16S rRNA gene in the same species

The *R. rhodochrous* ATCC BAA-870 genome encodes four 16S rRNA genes, consistent with the average 16S gene count statistics of *Rhodococcus* genomes. From a search of The Ribosomal RNA Database, of the 28 *Rhodococcus* genome records deposited in the NCBI database, 16S rRNA gene counts range from 3 to 5 copies, with an average of 4 [43]. Of the four 16S rRNA genes found in *R. rhodochrous* ATCC BAA-870, two pairs are identical (i.e. there are two copies of two different 16S rRNA genes). One of each identical 16S rRNA gene was used in nucleotide-nucleotide BLAST for highly similar sequences [44]. BLAST results (complete sequences with percentage identity greater than 95.5%) were used for comparison of *R. rhodochrous* ATCC BAA-870 to other similar species using 16S rRNA multiple sequence alignment and phylogeny in ClustalO and ClustalW respectively [45–47] (Fig. 1). Nucleotide BLAST results of the two different *R. rhodochrous* ATCC BAA-870 16S rRNA genes show closest sequence identities to *Rhodococcus* sp. 2G and *R. pyridinovorans* SB3094, with either 100% or 99.74% identities to both strains depending on the 16S rRNA copy.

We used the in silico DNA-DNA hybridisation tool, the Genome-to-Genome Distance Calculator (GGDC) version 2.1 [48–50], to assess the genome similarity of *R. rhodochrous* ATCC BAA-870 to its closest matched strains based on 16S rRNA alignment (*R. pyridinovorans* SB3094 and *Rhodococcus* sp. 2G). The results of genome based species and subspecies delineation, and difference in GC content, is summarised (Additional file 1: Table S3), with *R. jostii* RHA1 additionally shown for comparison. GC differences of below 1% would indicate the same species, and therefore *R. rhodochrous* ATCC BAA-870 cannot be distinguished from the other strains based on GC content. Digital DNA-DNA hybridisation values of more than 70 and 79% are the threshold for delineating type strains and subspecies. While 16S rRNA sequence alignment and GC content suggest that *R. rhodochrous* ATCC BAA-870 and *R. pyridinovorans* SB3094 and *Rhodococcus* sp. 2G are closely related strains, the GGDC supports their delineation at the subspecies level.

### Genome annotation

**Fig. 2:**
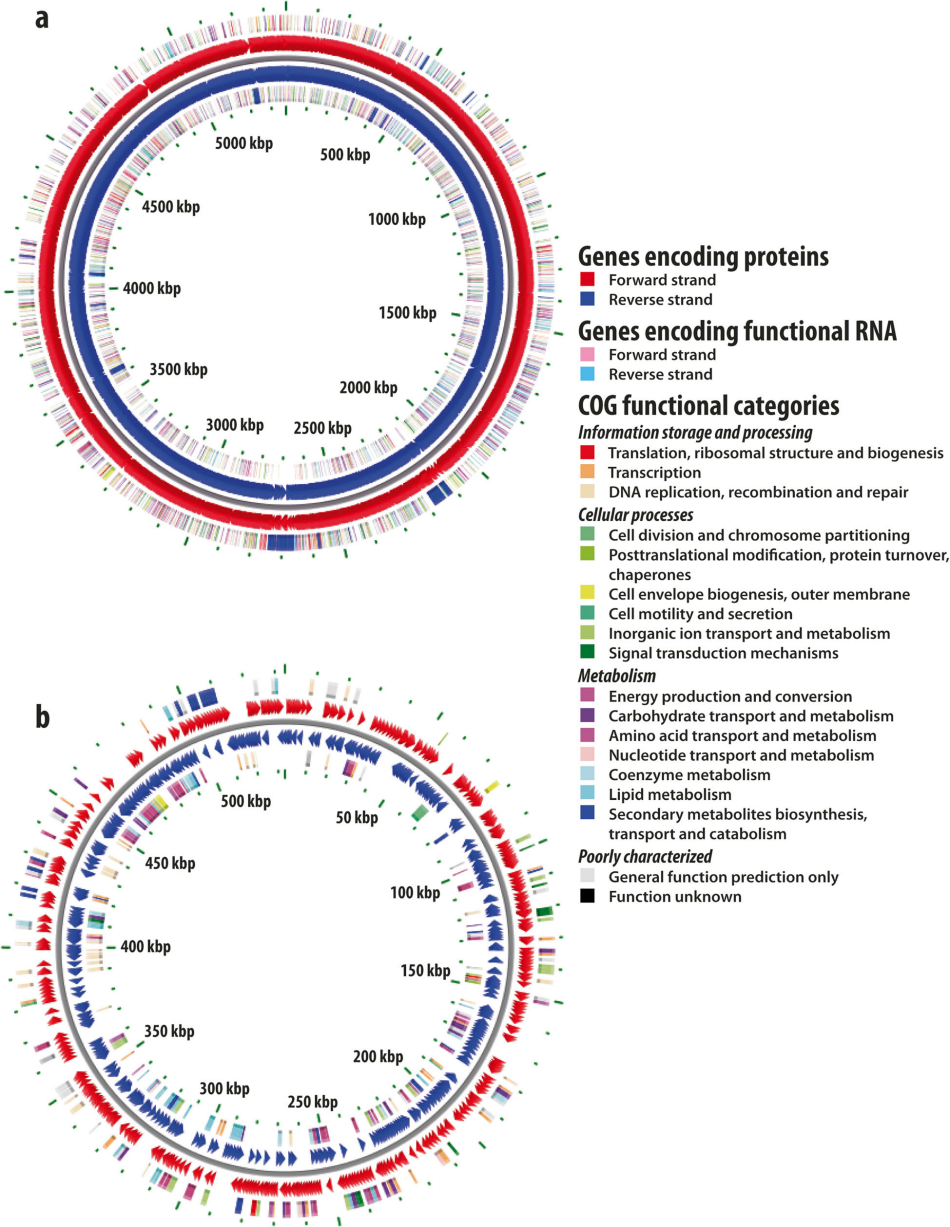
BASys bacterial annotation summary view of the *Rhodococcus rhodochrous* ATCC BAA-870 genome. BASys visual representation of **a** the 5,370,537 bp chromosome, with a breakdown of the 6871 genes encoded, and **b** the 533,288 bp linear plasmid, with a breakdown of the 677 genes encoded. Different colours indicate different subsystems for catabolic and anabolic routes

**Fig. 3:**
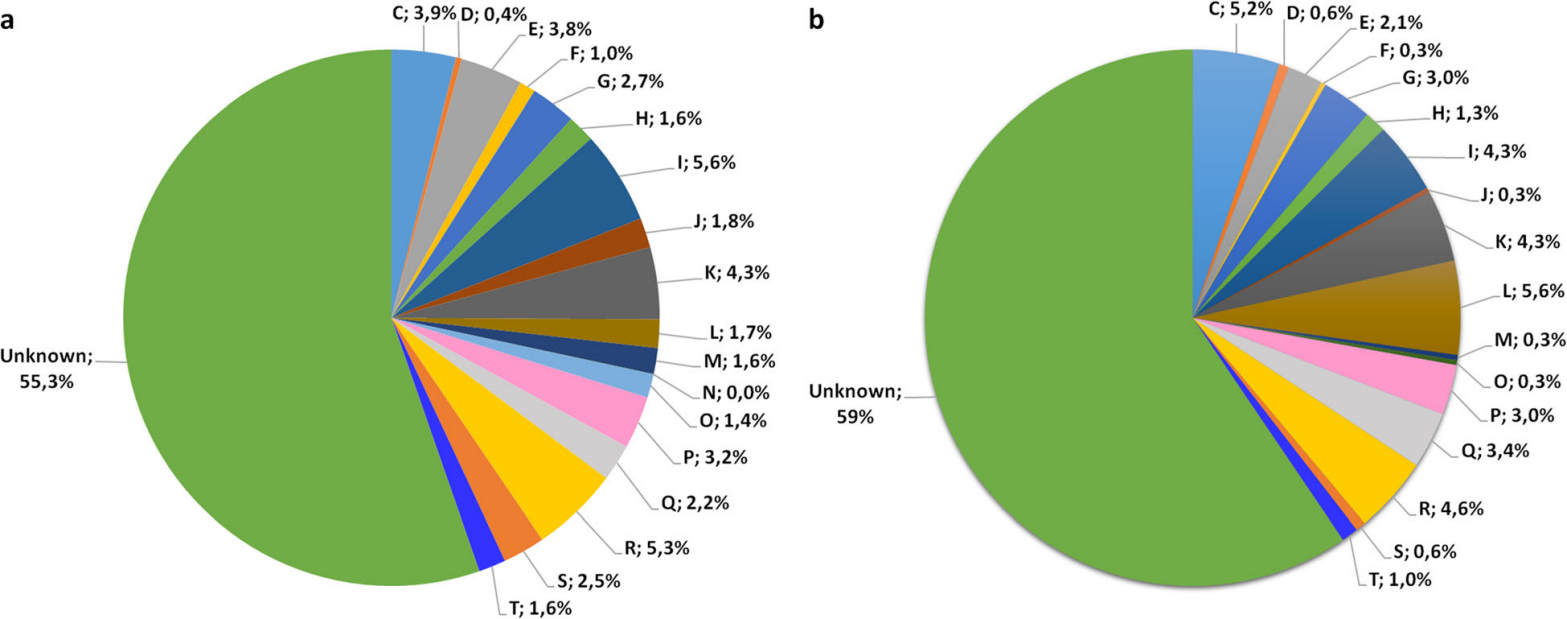
Protein function breakdown of *Rhodococcus rhodochrous* ATCC BAA-870 based on BASys annotation COG classifications. Unknown proteins form the majority of proteins in the BASys annotated genome, and make up 55 and 59% respectively of genes in the **a** chromosome and **b** plasmid. For simplicity, functional categories less than 0.02% are not included in the graphic. Letters refer to COG functional categories, with one-letter abbreviations: C - Energy production and conversion; D - Cell division and chromosome partitioning; E - Amino acid transport and metabolism; F - Nucleotide transport and metabolism; G - Carbohydrate transport and metabolism; H - Coenzyme metabolism; I - Lipid metabolism; J - Translation, ribosomal structure and biogenesis; K - Transcription; L - DNA replication, recombination and repair; M - Cell envelope biogenesis, outer membrane; N - Secretion, motility and chemotaxis; O - Posttranslational modification, protein turnover, chaperones; P - Inorganic ion transport and metabolism; Q - Secondary metabolites biosynthesis, transport and catabolism; R - General function prediction only; S - COG of unknown function; T - Signal transduction mechanisms

The assembled genome sequence of *R. rhodochrous* ATCC BAA-870 was submitted to the Bacterial Annotation System web server, BASys, for automated, in-depth annotation [51]. The BASys annotation was performed using raw sequence data for both the chromosome and plasmid of *R. rhodochrous* ATCC BAA-870 with a total genome length of 5.9 Mbp, in which 7548 genes were identified and annotated (Fig. 2, Table 1). The plasmid and chromosome encode a predicted 677 and 6871 genes, respectively. 56.9% of this encodes previously identified proteins of unknown function and includes 305 conserved hypothetical proteins. A large proportion of genes are labelled ‘hypothetical’ based on sequence similarity and/or the presence of known signature sequences of protein families (Fig. 3). Out of 7548 BASys annotated genes, 1481 are annotated enzymes that could be assigned an EC number (20%). Confirmation of annotation was performed manually for selected sequences. In BASys annotation, COGs (Clusters of Orthologous Groups) were automatically delineated by comparing protein sequences encoded in complete genomes representing major phylogenetic lineages [52]. As each COG consists of individual proteins or groups of paralogs from at least 3 lineages, it corresponds to an ancient conserved domain [53, 54]. A total of 3387 genes annotated in BASys were assigned a COG function (44.9% of annotated genes), while 55 and 59% of annotated genes on the chromosome and plasmid respectively have unknown function.

**Fig. 4:**
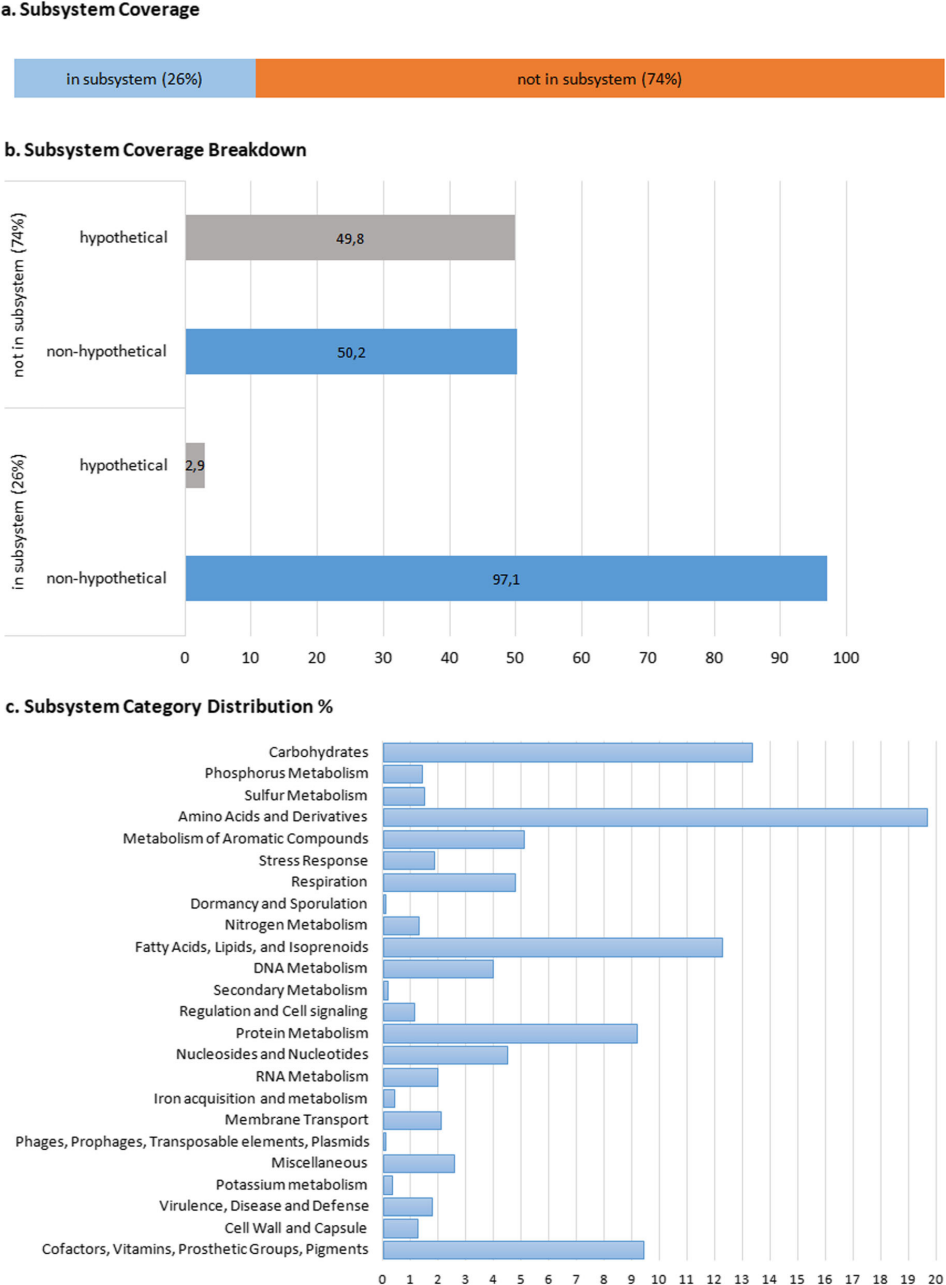
RAST annotation summary of the *Rhodococcus rhodochrous* ATCC BAA-870 genome. RAST annotation results show **a** the subsystem coverage, **b** the subsystem coverage breakdown, and **c** organisation of the subsystems by cellular process as a percentage showing the distribution of annotations across defined structural and functional subsystem roles. RAST uses a subsystem approach, in which annotations are assigned to groups with similar functional or structural roles. For *R. rhodochrous* ATCC BAA-870, 26% of annotated genes belong to an identified functional role, or subsystem. The coverage breakdown shows the percentage of hypothetical and non-hypothetical annotations for genes assigned to subsystems and those for which a known functional role is not assigned (i.e. those not in the subsystem)

The genome sequence run through RAST (Rapid Annotation using Subsystem Technology) predicted fewer (5535) protein coding sequences than BASys annotation (Fig. 4), showing the importance of the bioinformatics tool used. The RAST subsystem annotations are assigned from the manually curated SEED database, in which hypothetical proteins are annotated based only on related genomes. RAST annotations are grouped into two sets (genes that are either in a subsystem, or not in a subsystem) based on predicted roles of protein families with common functions. Genes belonging to recognised subsystems can be considered reliable and conservative gene predictions. Annotation of genes that do not belong to curated protein functional families however (i.e. those not in the subsystem), may be underpredicted by RAST, since annotations belonging to subsystems are based only on related neighbours. Based on counts of total genes annotated in RAST (5535), only 26% are classified as belonging to subsystems with known functional roles, while 74% of genes do not belong to known funtional roles. Overall 38% of annotated genes were annotated as hypothetical irrespective of whether or not they were included in subsystems. The use of two genome annotation pipelines allowed us to manually compare and search for enzymes, or classes of enzymes, using both the subsystem based, known functional pathway categories provided by RAST (Fig. 4), as well as the COG classification breakdowns provided by BASys (Fig. 3 and Additional file 1: Table S4). From both the RAST and BASys annotated gene sets, several industrially relevant enzyme classes are highlighted and discussed further in the text.

The average GC content of the *R. rhodochrous* ATCC BAA-870 chromosome and plasmid is 68.2 and 63.8%, respectively. The total genome has a 90.6% coding ratio, and on average large genes, consisting of ~ 782 bps per gene. Interestingly, the distribution of protein lengths on the chromosome is bell-shaped with a peak at 350 bps per gene, while the genes on the plasmid show two size peaks, one at 100 bps and one at 350 bps.

### Transcriptional control

Transcriptional regulatory elements in *R. rhodochrous* ATCC BAA-870 include 18 sigma factors, at least 8 regulators of sigma factor, and 118 other genes involved in signal transduction mechanisms (COG T), 261 genes encoding transcriptional regulators and 47 genes encoding two-component signal transduction systems. There are 129 proteins in *R. rhodochrous* ATCC BAA-870 associated with translation, ribosomal structure and biogenesis (protein biosynthesis). The genome encodes all ribosomal proteins, with the exception of S21, as occurs in other actinomycetes. RAST annotation predicts 66 RNAs. The 56 tRNAs correspond to all 20 natural amino acids and include two tRNA^fMet^. Additional analysis of the genome sequence using the tRNA finding tool tRNAScan-SE v. 2.0 [55, 56] confirms the presence of 56 tRNA genes in the *R. rhodochrous* ATCC BAA-870 genome, made up of 52 tRNA genes encoding natural amino acids, 2 pseudogenes, one tRNA with mismatched isotype and one + 9 Selenocysteine tRNA.

### Protein location in the cell

It is often critical to know where proteins are located in the cell in order to understand their function [57], and prediction of protein localization is important for both drug targeting and protein annotation. In this study, prediction was done using the BASys SignalP signal prediction service [51]. The majority of annotated proteins are soluble and located in the cytoplasm (83%), while proteins located at the cellular membrane make up 16% of the total. Cell membrane proteins include proteins that form part of lipid anchors, peripheral and integral cell membrane components, as well as proteins with single or multiple pass functions. Of the membrane proteins in *R. rhodochrous* ATCC BAA-870, 47% constitute single-pass, inner or peripheral membrane proteins, while 41% are multi-pass membrane proteins. Most of the remaining proteins will be transported over the membrane. The periplasm contains proteins distinct from those in the cytoplasm which have various functions in cellular processes, including transport, degradation, and motility. Periplasmic proteins would mostly include hydrolytic enzymes such as proteases and nucleases, proteins involved in binding of ions, vitamins and sugar molecules, and those involved in chemotaxic responses. Detoxifying proteins, such as penicillin binding proteins, are also presumed to be located mostly in the periplasm.

### Transport and metabolism

A total of 1504 genes are implicated in transport. Numerous components of the ubiquitous transporter families, the ATP-Binding Cassette (ABC) superfamily and the Major Facilitator Superfamily (MFS), are present in *Rhodococcus* strain BAA-870. MFS transporters are single-polypeptide secondary carriers capable only of transporting small solutes in response to chemiosmotic ion gradients [58, 59]. *R. rhodochrous* ATCC BAA-870 has 81 members of the MFS, mostly from the phthalate permease and sugar transporter families. There are dozens of families within the ABC superfamily, and each family generally correlates with substrate specificity. Transporters of *R. rhodochrous* ATCC BAA-870 include at least 122 members of the ABC superfamily, which includes both uptake and efflux transport systems. Out of 3387 genes assigned a COG function, 1486 (44%) are associated with transport and metabolism. These include 206 carbohydrate, 271 amino acid, 121 coenzyme, 236 inorganic ion, 411 lipid and 67 nucleotide transport and metabolism gene functions, and 174 secondary metabolite biosynthesis, transport and catabolism genes.

The complete biosynthetic pathways for all nucleotides, nucleosides and natural amino acids are also contained in the genome of *R. rhodochrous* ATCC BAA-870. The central metabolism of strain BAA-870 includes glycolysis, gluconeogenesis, the pentose phosphate pathway, and the tricarboxylic acid cycle, a typical metabolic pathway for an aerobic organism. There is no evidence for the Entner-Doudoroff pathway (including 6-phosphogluconate dehydratase and 2-keto-3-deoxyphosphogluconate aldolase) in *R. rhodochrous* ATCC BAA-870. General metabolic enzymes such as lipases and esterases [60, 61] are, however, present in this strain.

### Aromatic catabolism and oxidoreductases

As deduced from the better characterized pseudomonads [62], a large number of ‘peripheral aromatic’ pathways funnel a broad range of natural and xenobiotic compounds into a restricted number of ‘central aromatic’ pathways. Analysis of the *R. rhodochrous* ATCC BAA-870 genome suggests that at least four major pathways exist for the catabolism of central aromatic intermediates. The dominant portion of annotated enzymes is involved in oxidation and reduction, which is typical for catabolism. There are about 500 oxidoreductase related genes including oxidases, hydrogenases, reductases, oxygenases, dioxygenases, cytochrome P450s, catalases and peroxiredoxins. Furthermore, there are 71 monooxy-genase genes, 11 of which are on the plasmid.

In *R. rhodochrous* ATCC BAA-870 there are 14 cytochrome P450 genes and 87 oxygenase genes. It is unclear which oxygenases are catabolic and which are involved in secondary metabolism. Oxygenase genes include three cyclopentanone monooxygenases (EC 1.14.13.16) and a phenol monooxygenase (EC 1.14.13.7) on the plasmid, a methane monooxy-genase (EC 1.14.13.25), two alkane 1-monooxygenases (EC 1.14.15.3) and five phenylacetone monooxygenases (EC 1.14.13.92), one of which is on the plasmid.

### Nitrile biocatalysis

Rhodococci are well known for their application in the commercial manufacture of amides and acids through hydrolysis of the corresponding nitriles. *R. rhodochrous* J1 can convert acrylonitrile to the commodity chemical acrylamide [63], and both Mitsubishi Rayon Co., Ltd. (Japan) and Senmin (South Africa) are applying this biocatalytic reaction at the multi-kiloton scale. Lonza Guangzhou Fine Chemicals use the same biocatalyst for large-scale commercial synthesis of nicotinamide from 3-cyanopyridine [64]. Both processes rely on rhodococcal nitrile hydratase activity [65].

As *R. rhodochrous* ATCC BAA-870 was isolated from a nitrile enrichment culture [33], we were very interested in its nitrile degrading enzymes. As expected, strain BAA-870 contains several nitrile converting enzymes: a low molecular weight cobalt-containing nitrile hydratase and two nitrilases, along with several amidases. The low molecular weight nitrile hydratase and two amidase genes form a cluster, along with their associated regulatory elements, including cobalt transport genes necessary for uptake of cobalt for inclusion in the nitrile hydratase active site. Interestingly, this cluster is found on the plasmid. The alternative nitrile hydrolysis enzyme, nitrilase, is also found in *R. rhodochrous* ATCC BAA-870. It expresses an enantioselective aliphatic nitrilase encoded on the plasmid, which is induced by dimethylformamide [37]. Another nitrilase/cyanide hydratase family protein is also annotated on the plasmid (this study) but has not been characterised.

### Secondary metabolism and metabolite biosynthesis clusters

The ongoing search for new siderophores, antibiotics and antifungals has led to a recent explosion of interest in mining bacterial genomes [66], and the secondary metabolism of diverse soil-dwelling microbes remains relatively underexplored despite their huge biosynthetic potential [67]. Evidence of an extensive secondary metabolism in *R. rhodochrous* ATCC BAA-870 is supported by the presence of at least 227 genes linked to secondary metabolite biosynthesis, transport and catabolism. The genome contains 15 biosynthetic gene clusters associated with secondary metabolites or antibiotics, identified by antiSMASH (antibiotics and Secondary Metabolite Analysis Shell pipeline, version 5.0.0) [68, 69]. Biosynthetic gene clusters identified in *R. rhodochrous* BAA-870 include ectoine (1,4,5,6-tetrahydro-2-methyl-4-pyrimidinecarboxylic acid), butyrolactone, betalactone, and type I polyketide synthase (PKS) clusters, as well as three terpene and seven nonribosomal peptide synthetase (NRPS) clusters. An additional six putative biosynthetic clusters were identified on the *R. rhodochrous* ATCC BAA-870 plasmid, four of an unknown type, and the other two with low similarity to enterobactin and lipopolysaccharide biosynthetic clusters.

Soil-dwelling rhodococci present rich possible sources of terpenes and isoprenoids which are implicated in diverse structural and functional roles in nature. AntiSMASH analysis revealed 3 terpene biosynthetic clusters in the genome of *R. rhodochrous* ATCC BAA-870. Some of the examples of annotated *R. rhodochrous* ATCC BAA-870 genes related to terpene and isoprenoid biosynthesis include phytoene saturase and several phytoene synthases, dehydrogenases and related proteins, as well as numerous diphosphate synthases, isomerases and epimerases. The genome also contains lycopene cyclase, a novel non-redox flavoprotein [70], farnesyl diphosphate synthase, farnesyl transferase, geranylgeranyl pyrophosphate synthetases and digeranylgeranylglycerophospholipid reductase. Farnesyl diphosphate synthase and geranylgeranyl pyrophosphate synthases are potential anticancer and anti-infective drug targets [71]. In addition, the *R. rhodochrous* ATCC BAA-870 plasmid encodes a lactone ring-opening enzyme, monoterpene epsilon-lactone hydrolase.

The *R. rhodochrous* ATCC BAA-870 genome has two PKS genes, one regulator of PKS expression, one exporter of polyketide antibiotics, as well as three polyketide cyclase/dehydrases involved in polyketide biosynthesis. In addition, there are two actinorhodin polyketide dimerases. A total of five NRPS genes for secondary metabolite synthesis can be found on the chromosome. *R. rhodochrous* ATCC BAA-870 contains 4 probable siderophore-binding lipoproteins, 3 probable siderophore transport system permeases, and two probable siderophore transport system ATP-binding proteins. Other secondary metabolite genes found in *R. rhodochrous* ATCC BAA-870 include a dihydroxybenzoic acid-activating enzyme (2,3-dihydroxybenzoate-AMP ligase bacillibactin siderophore), phthiocerol/phenolphthiocerol synthesis polyketide synthase type I, two copies of linear gramicidin synthase subunits C and D genes, and tyrocidine synthase 2 and 3.

### CRISPR

One putative clustered regularly interspaced short palindromic repeat (CRISPR) is contained in the *R. rhodochrous* ATCC BAA-870 genome, according to analysis by CRISPRCasFinder [72]. Associated CRISPR genes are not automatically detected by the CRISPRCasFinder tool, but manual searches of the annotated genome for Cas proteins reveal possible Cas9 candidate genes within the *R. rhodochrous* ATCC BAA-870 genome, including a *ruv*C gene, and HNH endonuclease and nuclease genes.

### Horizontal gene transfer

Organisms acquire diverse metabolic capacity through gene duplications and acquisitions, typically mediated by transposases. Analysis using IslandViewer (for computational identification of genomic islands) [73] identifies 10 possible large genomic island regions in *R. rhodochrous* ATCC BAA-870 which may have been obtained through horizontal mobility. Half of these genomic islands are located on the plasmid and make up 90% of the plasmid coding sequence. The low molecular weight cobalt-containing nitrile hydratase operon is located on an 82.5 kbp genomic island that includes 57 predicted genes in total. Other genes of interest located on this same genomic island include crotonase and enoyl-CoA hydratase, 10 dehydrogenases including four acyl-CoA dehydrogenases and two aldehyde dehydrogenases, four hydrolases including 5-valerolactone hydrolase and amidohydrolase, beta-mannosidase, haloacid dehalogenase and five oxidoreductases. The *R. rhodochrous* ATCC BAA-870 genome contains 31 transposase genes found in the genomic regions identified by IslandViewer, one of which is from the IS30 family, a ubiquitous mobile insertion element in prokaryotic genomes [74]. Other transposase genes belonging to at least 10 different families of insertion sequences were identified in *R. rhodochrous* ATCC BAA-870, including ISL3, IS5, IS701, two IS1634, three IS110, three IS3, three IS256, five IS21, and six IS630 family transposases. The majority of these transposons (27 of the 31 identified by IslandViewer) are located on the plasmid.

## Discussion

### Sequencing and annotation

New sequencing technology has revolutionized the cost and pace of obtaining genome information, and there has been a drive to sequence the genomes of organisms which have economic applications, as well as those with environmental interest [75, 76]. This holds true for *Rhodococcus* genomes, of which only two were sequenced in 2006, while 13 years later 353 genomes are now available, mainly due to Whole Genome Shotgun sequencing efforts (Additional file 1: Table S1). The impact of better and faster sequencing, using improved sequencing techniques, is evident in this case of sequencing the *R. rhodochrous* ATCC BAA-870 genome: an initial assembly of a 36-cycle, single-ended Illumina library sequence performed in 2009, together with a mate-pair library, yielded a 6 Mbp genome of 257 scaffolds. A more recently performed paired-end Illumina library combined with the previous mate-pair library reduced this to only 6 scaffolds (5.88 Mbp), showing the improved second-generation sequencing results in only 10 years’ time. The presence of four copies of 16S-like genes was the main reason for the assembly to break into 6 scaffolds. Using third generation sequencing (Nanopore), this problem was overcome, and the genome could be fully assembled. Hence, we see second generation sequencing evolving to produce higher quality assemblies, but the combination with 3rd generation sequencing was necessary to obtain the full-length closed bacterial genome.

It has been assumed that the annotation of prokaryotic genomes is simpler than that of the intron-containing genomes of eukaryotes. However, annotation has been shown to be problematic, especially with over- or under-prediction of small genes where the criterion used to decide the size of an open reading frame (ORF) can systematically exclude annotation of small proteins [77]. Warren et al. 2010, used high performance computational methods to show that current annotated prokaryotic genomes are missing 1153 candidate genes that have been excluded from annotations based on their size [77]. These missing genes do not show strong similarities to gene sequences in public databases, indicating that they may belong to gene families which are not currently annotated in genomes. Furthermore, they uncovered ~ 38,895 intergenic ORFs, currently labelled as ‘putative’ genes only by similarity to annotated genes, meaning that the annotations are absent. Therefore, prokaryotic gene finding and annotation programs do not accurately predict small genes, and are limited to the accuracy of existing database annotations. Hypothetical genes (genes without any functional assignment), genes that are assigned too generally to be of use, misannotated genes and undetected real genes remain the biggest challenges in assigning annotations to new genome data [78–81]. As such, there is the possibility that we are under-estimating the number of genes present on this genome.

Apart from possible misannotation, the algorithm or software used for the annotation plays a huge role in the outcome. In this research both BASys (Fig. 2) and RAST (Fig. 4) were used as annotation tools, resulting in 7548 and 5535 predicted genes respectively. BASys annotation may provide an overprediction of gene numbers, due to sensitive GLIMMER ab initio gene prediction methods that can give false positives for higher GC content sequences [82]. This shows the importance of the bioinformatics tool used, which makes comparison to other genomes more difficult.

### Size and content of the genome

The genomic content of *R. rhodochrous* ATCC BAA-870 was outlined and compared to other rhodococcal genomes. Sequences of other *Rhodococcus* genomes were obtained from the Genome database at NCBI [83] and show a large variation in genome size between 4 and 10 Mbp (Additional file 1: Table S1), with an average of 6.1 ± 1.6 Mbp. The apparent total genome size of *R. rhodochrous* ATCC BAA-870, 5.9 Mbp (consisting of a 5.37 Mbp genome and a 0.53 Mbp plasmid), is close to the average. From the well-described rhodococci (Table 1), the genome of *R. jostii* RHA1 is the largest rhodococcal genome sequenced to date (9.7 Mbp), but only 7.8 Mbp is chromosomal, while the pathogenic *R. hoagii* genomes are the smallest at ~ 5 Mbp. All rhodococcal genomes have a high GC content, ranging from 62 to 71%. The average GC content of the *R. rhodochrous* ATCC BAA-870 chromosome and plasmid is 68.2 and 63.8%, respectively. *R. jostii* RHA1 has the lowest percentage coding DNA (87%), which is predictable given its large overall genome size, while *R. rhodochrous* ATCC BAA-870 has a 90.6% coding ratio, which is in line with its smaller total size. Interestingly, the distribution of protein lengths on the chromosome is different from those on the plasmid. Together with the lower GC content, this shows that the plasmid content was probably acquired over different occasions [84].

### Fundamental and applicable biocatalytic properties of rhodococci

Catabolism typically involves oxidative enzymes. The presence of multiple homologs of catabolic genes in all *Rhodococcus* species suggests that they may provide a comprehensive biocatalytic profile [1]. *R. rhodochrous* ATCC BAA-870 combines this with multiple transport systems (44% of total COG annotated genes), highlighting the metabolic versatility of this *Rhodococcus*, which facilitates the use of whole cells in biotechnological applications.

McLeod et al. reported that *R. jostii* RHA1 contains genes for the Entner-Doudoroff pathway (which requires 6-phosphogluconate dehydratase and 2-keto-3-deoxyphosphogluconate aldolase to create pyruvate from glucose) [10]. The Entner-Doudoroff pathway is, however, rare in Gram positive organisms which preferably use glycolysis for a richer ATP yield. There is no evidence of this pathway existing in *R. rhodochrous* ATCC BAA-870, indicating that it is not a typical rhodococcal trait, but the RHA1 strain must have acquired it rather recently.

Analysis of the *R. rhodochrous* ATCC BAA-870 genome suggests that at least four major pathways exist for the catabolism of central aromatic intermediates, comparable to the well-defined aromatic metabolism of *Pseudomonas putida* KT2440 strain [85]. In *R. rhodochrous* ATCC BAA-870 the dominant portion of annotated enzymes are involved in oxidation and reduction. There are about 500 oxidoreductase related genes, which is quite a high number compared to other bacteria of the same size, but in line with most other (sequenced) rhodococci [86]. *Rhodococcus* genomes usually encode large numbers of oxygenases [1], which is also true for strain BAA-870 (71). Some of these are flavonoid proteins with diverse useful activities [87], which includes monooxygenases capable of catalysing Baeyer–Villiger oxidations wherein a ketone is converted to an ester [88, 89].

The 14 cytochrome P450 genes in *R. rhodochrous* ATCC BAA-870 reflects a fundamental aspect of rhodococcal physiology. Similarly, the number of cytochrome P450 genes in *R. jostii* RHA1 is 25 (proportionate to the larger genome) which is typical of actinomycetes. Although it is unclear which oxygenases in *R. rhodochrous* ATCC BAA-870 are catabolic and which are involved in secondary metabolism, their abundance is consistent with a potential ability to degrade an exceptional range of aromatic compounds (oxygenases catalyse the hydroxylation and cleavage of these compounds). Rhodococci are well known to have the capacity to catabolise hydrophobic compounds, including hydrocarbons and polychlorinated biphenyls (PCBs), mediated by a cytochrome P450 system [90–93]. Cytochrome P450 oxygenase is often found fused with a reductase, as in *Rhodococcus* sp. NCIMB 9784 [94]. Genes associated with biphenyl and PCB degradation are found in multiple sites on the *R. jostii* RHA1 genome, both on the chromosome as well as on linear plasmids [1]. *R. jostii* RHA1 was also found to show lignin-degrading activity, possibly based on the same oxidative capacity as that used to degrade biphenyl compounds [95].

The oxygenases found in rhodococci include multiple alkane monooxygenases (genes *alkB1*–*alkB4*) [96], steroid monooxygenase [97], styrene monooxygenase [98], peroxidase [99] and alkane hydroxylase homologs [100]. *R. rhodochrous* ATCC BAA-870 has 87 oxygenase genes while the PCB degrading *R. jostii* RHA1 has 203 oxygenases, including 19 cyclohexanone monooxygenases (EC 1.14.13.22), implying that of the two, strain BAA-870 is less adept at oxidative catabolism. Rhodococcal cyclohexanone monooxygenases can be used in the synthesis of industrially interesting compounds from cyclohexanol and cyclohexanone. These include adipic acid, caprolactone (for polyol polymers) and 6-hydroxyhexanoic acid (for coating applications) [65]. Chiral lactones can also be used as intermediates in the production of prostaglandins [101]. The same oxidative pathway can be used to biotransform cyclododecanone to lauryl lactone or 12-hydroxydodecanoic acid [102, 103]. Cyclododecanone monooxygenase of *Rhodococcus* SC1 was used in the kinetic resolution of 2-substituted cycloketones for the synthesis of aroma lactones in good yields and high enantiomeric excess [104]. Similar to *R. jostii* RHA1, *R. rhodochrous* ATCC BAA-870 encodes several monooxygenases. All these redox enzymes could be interesting for synthetic purposes in industrial biotechnological applications.

The presence of an ectoine biosynthesis cluster suggests that *R. rhodochrous* ATCC BAA-870 has effective osmoregulation and enzyme protection capabilities. *R. rhodochrous* ATCC BAA-870, together with other *Rhodococcus* strains, is able to support diverse environments and can tolerate harsh chemical reactions when used as whole cell biocatalysts, and it is likely that ectoine biosynthesis plays a role in this. Regulation of cytoplasmic solute concentration through modulation of compounds such as inorganic ions, sugars, amino acids and polyols provides a versatile and effective osmo-adaptation strategy for bacteria in general. Ectoine and hydroxyectoine are common alternate osmoregulation solutes found especially in halophilic and halotolerant microorganisms [105, 106], and hydroxyectoine has been shown to confer heat stress protection in vivo [107]. Ectoines provide a variety of useful biotechnological and biomedical applications [108], and strains engineered for improved ectoine synthesis have been used for the industrial production of hydroxyectoine as a solute and enzyme stabiliser [109, 110]. The special cell-wall structure of rhodococci might make these organisms a better choice as production organism.

Terpenes and isoprenoids provide a rich pool of natural compounds with applications in synthetic chemistry, pharmaceutical, flavour, and even biofuel industries. The structures, functions and chemistries employed by the enzymes involved in terpene biosynthesis are well known, especially for plants and fungi [71, 111]. However, it is only recently that bacterial terpenoids have been considered as a possible source of new natural product wealth [112, 113], largely facilitated by the explosion of available bacterial genome sequences. Interestingly, bacterial terpene synthases have low sequence similarities, and show no significant overall amino acid identities compared to their plant and fungal counterparts. Yamada et al. used a genome mining strategy to identify 262 bacterial synthases, and subsequent isolation and expression of genes in a *Streptomyces* host confirmed the activities of these predicted genes and led to the identification of 13 previously unknown terpene structures [112]. The three biosynthetic clusters annotated in strain BAA-870 may therefore be an underrepresentation of possible pathways for these valuable compounds.

A total of five NRPS genes for secondary metabolite synthesis can be found on the chromosome, which is not much compared to *R. jostii* RHA1 that contains 24 NRPS and seven PKS genes [10]. Like strain ATCC BAA-870, *R. jostii* RHA1 was also found to possess a pathway for the synthesis of a siderophore [114]. The multiple PKS and NRPS clusters suggest that *R. rhodochrous* ATCC BAA-870 may host a significant potential source of molecules with immunosuppressing, antifungal, antibiotic and siderophore activities [115].

### Nitrile conversion

Many rhodococci can hydrolyse a wide range of nitriles [116–119]. The locations and numbers of nitrile converting enzymes in the available genomes of *Rhodococcus* were identified and compared to *R. rhodochrous* ATCC BAA-870 (Table 2). *R. rhodochrous* ATCC BAA-870 contains several nitrile converting enzymes which is in line with previous activity assays using this *Rhodococcus* strain [34, 35]. However, in most *R. rhodochrous* strains these enzymes are on the chromosome, while in *R. rhodochrous* ATCC BAA-870, they are found on a plasmid. In *R. rhodochrous* ATCC BAA-870 the nitrile hydratase is expressed constitutively, explaining why this strain is an exceptional nitrile biocatalyst [37]. Environmental pressure through chemical challenge by nitriles may have caused the elimination of regulation of the nitrile biocatalyst by transferring it to a plasmid.

**Table 2:** Comparison of nitrile converting enzymes in different *Rhodococcus* species

Organism	Nitrilase	Nitrile Hydratase	NHase regulators	Amidase	Amidase Regulators	NCBI Assembly Reference
*R. rhodochrous* ATCC BAA-870	2 (pl)	1 (pl)	4 (pl)	7 (chr) 2 (pl)	2 (pl)	this study
*R. erythropolis* PR4	–	1	4	12	1	GCF_000010105 [18]
*R. erythropolis* SK121	–	1	–	2	–	GCF_000174835 (no reference)
*R. hoagii* 103S	–	–	–	11	–	GCF_000196695 [17]
*R. hoagii* ATCC 33707	–	–	–	11	–	GCF_000164155 (no reference)
*R. jostii* RHA1	1	1	–	14 (chr) 1 (pl)	–	GCF_000014565 [10]
*R. opacus* B4	–	–	–	13	–	GCF_000010805 (no reference)
*R. opacus* PD630	–	2	–	13	2	GCF_000599555 GCF_000234335 [15, 16]
*Rhodococcus* sp. M8	–	2	1	9	–	GCF_001890475 [120]
*Rhodococcus* sp. YH3–3	1	2	1	13	1	GCF_001653035 [121]

Number of enzymes on the chromosome. If multiple enzymes are present on different genomic elements, the location is mentioned: *chr* chromosome or *pl* plasmids

The *R. jostii* RHA1 16S RNA sequence indicates that it is closely related to *R. opacus* [10] according to the taxonomy of Gürtler et al. (Fig. 1) [122]. *R. jostii* RHA1 expresses a nitrile hydratase (an acetonitrile hydratase) and utilises nitriles such as acetonitrile, acrylonitrile, propionitrile and butyronitrile [123], while *R. opacus* expresses nitrile hydrolysis activity [116]. *R. erythropolis* PR4 expresses a Fe-type nitrile hydratase [124], and *R. erythropolis* strains are well known for expressing this enzyme [116, 125, 126] as part of a nitrile metabolism gene cluster [122]. This enzyme has been repeatedly determined in this species from isolated diverse locations [127], expressing broad substrate profiles, including acetonitrile, propionitrile, acrylonitrile, butyronitrile, succinonitrile, valeronitrile, isovaleronitrile and benzonitrile [116].

The nitrile hydratase enzymes of *R. rhodochrous* have to date been shown to be of the Co-type [6, 126, 128], which are usually more stable than the Fe-type nitrile hydratases. They have activity against a broad range of nitriles, including phenylacetonitrile, 2-phenylpropionitrile, 2-phenylglycinonitrile, mandelonitrile, 2-phenylbutyronitrile, 3-phenylpropionitrile, *N*-phenylglycinonitrile, *p*-toluonitrile and 3-hydroxy-3-phenylpropionitrile [33]. *R. ruber* CGMCC3090 and other strains express nitrile hydratases [116, 129] while the nitrile hydrolysis activity of *R. hoagii* [116] is also attributed to a nitrile hydratase [130].

The alternative nitrile hydrolysis enzyme, nitrilase, is also common in rhodococci (Table 2), including *R. erythropolis* [131], *R. rhodochrous* [132–135], *R. opacus* B4 [136] and *R. ruber* [137, 138]. The nitrilase from *R. ruber* can hydrolyse acetonitrile, acrylonitrile, succinonitrile, fumaronitrile, adiponitrile, 2-cyanopyridine, 3-cyanopyridine, indole-3-acetonitrile and mandelonitrile [138]. The nitrilases from multiple *R. erythropolis* strains were active towards phenylacetonitrile [139]. *R. rhodochrous* nitrilase substrates include (among many others) benzonitrile for *R. rhodochrous* J1 [140] and crotononitrile and acrylonitrile for *R. rhodochrous* K22 [141]. *R. rhodochrous* ATCC BAA-870 expresses an enantioselective aliphatic nitrilase encoded on the plasmid, which is induced by dimethylformamide [37]. Another nitrilase/cyanide hydratase family protein is also annotated on the plasmid (this study) but has not been characterised. The diverse, yet sometimes very specific and enantioselective substrate specificities of all these rhodococci gives rise to an almost plug-and-play system for many different synthetic applications. Combined with their high solvent tolerance, rhodococci are very well suited as biocatalysts to produce amides for both bulk chemicals and pharmaceutical ingredients.

The large percentage of possible mobile genomic region making up the plasmid, together with the high number of transposon genes and the fact that the plasmid contains the machinery for nitrile degradation, strongly support our theory that *R. rhodochrous* ATCC BAA-870 has adapted its genome recently in response to the selective pressure of routine culturing in nitrile media in the laboratory. Even though isolated from contaminated soil, the much larger chromosome of *R. jostii* RHA1 in comparison has undergone relatively little recent genetic flux as supported by the presence of only two intact insertion sequences, relatively few transposase genes, and only one identified pseudogene [10]. The smaller *R. rhodochrous* ATCC BAA-870 genome, still has the genetic space and tools to adapt relatively easily in response to environmental selection.

### CRISPR

CRISPRs are unusual finds in rhodococcal genomes. Based on literature searches to date, only two other sequenced *Rhodococcus* strains were reported to contain potential CRISPRs. *R. opacus* strain M213, isolated from fuel-oil contaminated soil, has one confirmed and 14 potential CRISPRs [142], identified using the CRISPRFinder tool [143]. Pathak et al. also surveyed several other *Rhodococcus* sequences and found no other CRISPRs. Zhao and co-workers state that *Rhodococcus* strain sp. DSSKP-R-001, interesting for its beta-estradiol-degrading potential, contains 8 CRISPRs [144]. However, the authors do not state how these were identified. Pathak et al. highlight the possibility that the CRISPR in *R. opacus* strain M213 may have been recruited from *R. opacus* R7 (isolated from polycyclic aromatic hydrocarbon contaminated soil [145]), based on matching BLASTs of the flanking regions.

The *R. rhodochrous* ATCC BAA-870 CRISPR upstream and downstream regions (based on a 270- and 718 nucleotide length BLAST, respectively) showed significant, but not matching, alignment with several other *Rhodococcus* strains. The region upstream of the BAA-870 CRISPR showed a maximum 95% identity with that from *R. rhodochrous* strains EP4 and NCTC10210, while the downstream region showed 97% identities to *R. pyridinovorans* strains GF3 and SB3094, *R. biphenylivorans* strain TG9, and *Rhodococcus* sp. P52 and 2G. Analysis by PHAST phage search tool [146] identified the presence of 6 potential, but incomplete, prophage regions on the chromosome, and one prophage region on the plasmid, suggesting that the CRISPR acquisition in *R. rhodochrous* ATCC BAA-870 could also have arisen from bacteriophage infection during its evolutionary history.

### Identification of target genes for future biotechnology applications

An estimated 150 biocatalytic processes are currently being applied in industry [147–149]. The generally large and complex genomes of *Rhodococcus* species afford a wide range of genes attributed to extensive secondary metabolic pathways that are presumably responsible for an array of biotransformations and bioremediations. These secondary metabolic pathways have yet to be characterised and offer numerous targets for drug design as well as synthetic chemistry applications, especially since enzymes in secondary pathways are usually more promiscuous than enzymes in the primary pathways.

A number of potential genes which could be used for further biocatalyses have been identified in the genome of *R. rhodochrous* ATCC BAA-870. A substantial fraction of the genes have unknown functions, and these could be important reservoirs for novel gene and protein discovery. Most of the biocatalytically useful classes of enzyme suggested by Pollard and Woodley [150] are present on the genome: proteases, lipases, esterases, reductases, nitrilase/cyanohydrolase/nitrile hydratases and amidases, transaminase, epoxide hydrolase, monooxygenases and cytochrome P450s. Only oxynitrilases (hydroxynitrile lyases) and halohydrin dehalogenase were not detected, although a haloacid dehalogenase is present. Rhodococci are robust industrial biocatalysts, and the metabolic abilities of the *Rhodococcus* genus will continue to attract attention for industrial uses as further bio-degradative [6] and biopharmaceutical [151] applications of the organism are identified. Preventative and remediative biotechnologies will become increasingly popular as the demand for alternative means of curbing pollution increases and the need for new antimicrobial compounds and pharmaceuticals becomes a priority.

## Conclusions

The genome sequence of *R. rhodochrous* ATCC BAA-870 is one of 353 *Rhodococcus* genomes that are sequenced to date, but it is only the 4th sequence that has been fully characterised on a biotechnological level. Therefore, the sequence of the *R. rhodochrous* ATCC BAA-870 genome will facilitate the further exploitation of rhodococci for biotechnology applications, as well as enable further characterisation of a biotechnologically relevant organism. The genome has at least 1481 enzyme encoding genes, many of which have potential application in industrial biotechnology. Based on comparative annotation of the genome, up to 50% of annotated genes are hypothetical, while as much as 74% of genes may have unknown metabolic functions, indicating there is still a lot to learn about rhodococci.

## Methods

### Strain and culture conditions

*R. rhodochrous* ATCC BAA-870, isolated from industrial soil in Modderfontein, Johannesburg, South Africa, was grown routinely on Tryptone Soya Agar medium. For genomic DNA preparation, the strain was grown in 50 mL Tryptone Soya Broth overnight at 37 °C. Cells were centrifuged and the DNA purified using a Wizard® Genomic DNA Purification Kit (Promega, Madison, WI) or Ultraclean microbial DNA extraction kit (MoBio, Carlsbad, CA). DNA concentrations were measured spectrophotometrically by absorbance readings at 260 nm using a NanoDrop-1000 (Thermo Scientific, Wilmington, DE).

### Illumina sequencing

Genomic DNA of *R. rhodochrous* BAA-870 was used to obtain two libraries with different insert sizes. One 300 cycle paired-end library with insert-size of 550 bp was sequenced in-house on a MiSeq sequencer (Illumina, San Diego, CA) using TruSeq PCR-free library preparation. The second, a 50 cycle mate pair library with 5 kb insert-size, was performed at BaseClear (Leiden, The Netherlands). Data is available at NCBI under Bioproject accession number PRJNA487734.

### MinION sequencing

For Nanopore sequencing a 1D sequencing library (SQK-LSK108) was loaded onto a FLO-MIN106 (R9.4) flowcell, connected to the MinION Mk1B (Oxford Nanopore Technology, Oxford, United Kingdom). MinKNOW software (version 1.11.5; Oxford Nanopore) was used for quality control of active pores and for sequencing. Raw files generated by MinKNOW were base called, on a local compute server (HP ProLiant DL360 G9, 2x XEON E5-2695v3 14 Cores and 256 RAM), using Albacore (version 1.2.5; Oxford Nanopore). Reads, in fastq format, with a minimum length of 1000 bps were extracted, yielding 5.45 Gigabase sequence with an average read length of 9.09 kb.

### De novo assembly

De novo assembly was performed using Canu (v1.4, settings: genomesize = 6 m) [152] producing a 5.88 Mbp genome consisting of two contigs. One chromosome with a length of 5.35 Mbp, while the second covers a size of 0.531 Mbp which, based on the Canu assembly graph, is a linear plasmid. The paired-end Illumina library was aligned, using BWA [153], to the assembly and the resulting Binary Alignment Map file was processed by Pilon [154] for polishing the assembly (correcting assembly errors), using correction of only SNPs and short indels (−fix bases parameter).

### Annotation

The assembled genome sequence of *R. rhodochrous* ATCC BAA-870 was submitted to the Bacterial Annotation System web server, BASys, for automated, in-depth annotation of the chromosomal and plasmid sequences [51]. BASys annotates based on microbial ab initio gene prediction using GLIMMER [82]. The genome sequence was also run on the RAST (Rapid Annotation using Subsystem Technology) server using the default RASTtk annotation pipeline for comparison [155, 156]. RAST annotation uses the manually curated SEED database to infer gene annotations based on protein functional roles within families [157]. The two annotation pipelines offered different but useful and complimentary input formats and results, and gene annotations of interest could be manually compared and confirmed.

## Supplementary information

**Additional file 1: **SuppInfo Frederick et al. BAA-870 genome. **Table S1.** All sequenced *Rhodococcus* strains (353) according to the NCBI database (accessed 13/03/2019). **Table S2.** All complete sequenced *Rhodococcus* species ranked by release date according to the NCBI Genome database (accessed 11/03/2019). **Table S3.** Whole genome distance statistics between *Rhodococcus rhodochrous* ATCC BAA-870 and two closely matched strains. **Table S4.** *Rhodococcus rhodochrous* ATCC BAA-870 protein function breakdown based on BASys annotation COG classifications.

## Data Availability

The complete genome sequence of *R. rhodochrous* ATCC BAA 870 is deposited at NCBI GenBank, with Bioproject accession number PRJNA487734, and Biosample accession number SAMN09909133.

## Abbreviations

ABC: ATP-Binding Cassette
antiSMASH: Antibiotics and Secondary Metabolite Analysis Shell pipeline
BASys: Bacterial Annotation System
bps: Base pairs
COG: Cluster of Orthologous Groups
contig: Contiguous sequence
CRISPR: Clustered regularly interspaced short palindromic repeat
EC: Enzyme commission
GGDC: Genome-to-Genome Distance Calculator
Mbp: Megabase pairs
MFS: Major Facilitator Superfamily
NCBI: National Center for Biotechnology Information
NRPS: Nonribosomal peptide synthetase
ORF: Open reading frame
PCBs: Polychlorinated biphenyls
PKS: Polyketide synthase
RAST: Rapid Annotation using Subsystem Technology

## References

[1] van der Geize R, Dijkhuizen L (2004). Harnessing the catabolic diversity of rhodococci for environmental and biotechnological applications. Curr Opin Microbiol.

[2] Banerjee A, Sharma R, Banerjee UC (2002). The nitrile-degrading enzymes: current status and future prospects. Appl Microbiol Biotechnol.

[3] Yam KC, Geize R, Eltis LD, Alvarez HM (2010). Catabolism of aromatic compounds and steroids by *Rhodococcus*. Biology of Rhodococcus.

[4] Gray KA, Pogrebinsky OS, Mrachko GT, Xi L, Monticello DJ, Squires CH (1996). Molecular mechanisms of biocatalytic desulfurization of fossil fuels. Nat Biotech.

[5] Kobayashi M, Nagasawa T, Yamada H (1992). Enzymatic synthesis of acrylamide: a success story not yet over. Trends Biotechnol.

[6] Martínková L, Uhnáková B, Pátek M, Nešvera J, Křen V (2009). Biodegradation potential of the genus *Rhodococcus*. Environ Int.

[7] de Carvalho CC, Costa SS, Fernandes P, Couto I, Viveiros M (2014). Membrane transport systems and the biodegradation potential and pathogenicity of genus *Rhodococcus*. Front Physiol.

[8] Brady D. Biocatalytic hydrolysis of nitriles. In: Anastas PT, editor. Handbook of green chemistry, vol. 3. Weinheim: Wiley-VCH Verlag GmbH & Co. KGaA; 2010. p. 27–49.

[9] Sokolovská I, Rozenberg R, Riez C, Rouxhet PG, Agathos SN, Wattiau P (2003). Carbon source-induced modifications in the mycolic acid content and cell wall permeability of *Rhodococcus erythropolis* E1. Appl Environ Microbiol.

[10] McLeod MP, Warren RL, Hsiao WWL, Araki N, Myhre M, Fernandes C, Miyazawa D, Wong W, Lillquist AL, Wang D (2006). The complete genome of *Rhodococcus* sp. RHA1 provides insights into a catabolic powerhouse. Proc Natl Acad Sci U S A.

[11] Seto M, Kimbara K, Shimura M, Hatta T, Fukuda M, Yano K (1995). A novel transformation of polychlorinated biphenyls by *Rhodococcus* sp. strain RHA1. Appl Environ Microbiol.

[12] Masai E, Yamada A, Healy JM, Hatta T, Kimbara K, Fukuda M, Yano K (1995). Characterization of biphenyl catabolic genes of gram-positive polychlorinated biphenyl degrader *Rhodococcus* sp. strain RHA1. Appl Environ Microbiol.

[13] Sangal V, Goodfellow M, Jones AL, Schwalbe EC, Blom J, Hoskisson PA, Sutcliffe IC (2016). Next-generation systematics: an innovative approach to resolve the structure of complex prokaryotic taxa. Sci Rep.

[14] Kwasiborski A, Mondy S, Chong T-M, Chan K-G, Beury-Cirou A, Faure D (2015). Core genome and plasmidome of the quorum-quenching bacterium *Rhodococcus erythropolis*. Genetica.

[15] Chen Y, Ding Y, Yang L, Yu J, Liu G, Wang X, Zhang S, Yu D, Song L, Zhang H (2014). Integrated omics study delineates the dynamics of lipid droplets in *Rhodococcus opacus* PD630. Nuc Acids Res.

[16] Holder JW, Ulrich JC, DeBono AC, Godfrey PA, Desjardins CA, Zucker J, Zeng Q, Leach ALB, Ghiviriga I, Dancel C (2011). Comparative and functional genomics of *Rhodococcus opacus* PD630 for biofuels development. PLoS Genet.

[17] Letek M, González P, MacArthur I, Rodríguez H, Freeman TC, Valero-Rello A, Blanco M, Buckley T, Cherevach I, Fahey R (2010). The genome of a pathogenic *Rhodococcus*: Cooptive virulence underpinned by key gene acquisitions. PLoS Genet.

[18] Sekine M, Tanikawa S, Omata S, Saito M, Fujisawa T, Tsukatani N, Tajima T, Sekigawa T, Kosugi H, Matsuo Y (2006). Sequence analysis of three plasmids harboured in *Rhodococcus erythropolis* strain PR4. Environ Microbiol.

[19] Komukai-Nakamura S, Sugiura K, Yamauchi-Inomata Y, Toki H, Venkateswaran K, Yamamoto S, Tanaka H, Harayama S (1996). Construction of bacterial consortia that degrade Arabian light crude oil. J Ferment Bioeng.

[20] Chen B-S, Otten LG, Resch V, Muyzer G, Hanefeld U (2013). Draft genome sequence of *Rhodococcus rhodochrous* strain ATCC 17895. Stand Genomic Sci.

[21] Creason AL, Vandeputte OM, Savory EA, Davis EW, Putnam ML, Hu E, Swader-Hines D, Mol A, Baucher M, Prinsen E (2014). Analysis of genome sequences from plant pathogenic *Rhodococcus* reveals genetic novelties in virulence loci. PLoS One.

[22] Creason AL, Davis EW, Putnam ML, Vandeputte OM, Chang JH (2014). Use of whole genome sequences to develop a molecular phylogenetic framework for *Rhodococcus fascians* and the *Rhodococcus* genus. Front Plant Sci.

[23] Takai S, Hines SA, Sekizaki T, Nicholson VM, Alperin DA, Osaki M, Takamatsu D, Nakamura M, Suzuki K, Ogino N (2000). DNA sequence and comparison of virulence plasmids from *Rhodococcus equi* ATCC 33701 and 103. Infect Immun.

[24] Letek M, Ocampo-Sosa AA, Sanders M, Fogarty U, Buckley T, Leadon DP, González P, Scortti M, Meijer WG, Parkhill J (2008). Evolution of the *Rhodococcus equi vap* pathogenicity island seen through comparison of host-associated *vapA* and *vapB* virulence plasmids. J Bacteriol.

[25] Duquesne F, Hébert L, Sévin C, Breuil M-F, Tapprest J, Laugier C, Petry S (2010). Analysis of plasmid diversity in 96 *Rhodococcus equi* strains isolated in Normandy (France) and sequencing of the 87-kb type I virulence plasmid. FEMS Microbiol Lett.

[26] Francis I, De Keyser A, De Backer P, Simón-Mateo C, Kalkus J, Pertry I, Ardiles-Diaz W, De Rycke R, Vandeputte OM, El Jaziri M (2012). pFiD188, the linear virulence plasmid of *Rhodococcus fascians* D188. Mol Plant-Microbe Interact.

[27] Lessard P, O'Brien X, Currie D, Sinskey A (2004). pB264, a small, mobilizable, temperature sensitive plasmid from *Rhodococcus*. BMC Microbiol.

[28] Matsui T, Saeki H, Shinzato N, Matsuda H (2007). Analysis of the 7.6-kb cryptic plasmid pNC500 from *Rhodococcus rhodochrous* B-276 and construction of *Rhodococcus*–*E. coli* shuttle vector. Appl Microbiol Biotechnol.

[29] Na K-s, Nagayasu K, Kuroda A, Takiguchi N, Ikeda T, Ohtake H, Kato J (2005). Development of a genetic transformation system for benzene-tolerant *Rhodococcus opacus* strains. J Biosci Bioeng.

[30] Nakashima N, Tamura T (2004). Isolation and characterization of a rolling-circle-type plasmid from *Rhodococcus erythropolis* and application of the plasmid to multiple-recombinant-protein expression. Appl Environ Microbiol.

[31] De Mot R, Nagy I, De Schrijver A, Pattanapipitpaisal P, Schoofs G, Vanderleyden J (1997). Structural analysis of the 6 kb cryptic plasmid pFAJ2600 from *Rhodococcus erythropolis* NI86/21 and construction of *Escherichia coli*-*Rhodococcus* shuttle vectors. Microbiology.

[32] Stecker C, Johann A, Herzberg C, Averhoff B, Gottschalk G (2003). Complete nucleotide sequence and genetic organization of the 210-kilobase linear plasmid of *Rhodococcus erythropolis* BD2. J Bacteriol.

[33] Brady D, Beeton A, Zeevaart J, Kgaje C, van Rantwijk F, Sheldon RA (2004). Characterisation of nitrilase and nitrile hydratase biocatalytic systems. Appl Microbiol Biotechnol.

[34] Kinfe HH, Chhiba V, Frederick J, Bode ML, Mathiba K, Steenkamp PA, Brady D (2009). Enantioselective hydrolysis of β-hydroxy nitriles using the whole cell biocatalyst *Rhodococcus rhodochrous* ATCC BAA-870. J Mol Catal B Enzym.

[35] Chhiba V, Bode ML, Mathiba K, Kwezi W, Brady D (2012). Enantioselective biocatalytic hydrolysis of β-aminonitriles to β-amino-amides using *Rhodococcus rhodochrous* ATCC BAA-870. J Mol Catal B Enzym.

[36] Pawar SV, Yadav GD (2014). Enantioselective enzymatic hydrolysis of *rac*-mandelonitrile to *R*-mandelamide by nitrile hydratase immobilized on poly(vinyl alcohol)/chitosan–glutaraldehyde support. Ind Eng Chem Res.

[37] Chhiba-Govindjee VP, Mathiba K, van der Westhuyzen CW, Steenkamp P, Rashamuse JK, Stoychev S, Bode ML, Brady D (2018). Dimethylformamide is a novel nitrilase inducer in *Rhodococcus rhodochrous*. Appl Microbiol Biotechnol.

[38] Chen Jing, Zheng Ren-Chao, Zheng Yu-Guo, Shen Yin-Chu (2009). Microbial Transformation of Nitriles to High-Value Acids or Amides. Biotechnology in China I.

[39] Rodríguez JR (2014). Understanding nitrile-degrading enzymes: classification, biocatalytic nature and current applications. Rev Latinoam Biotecnol Ambient Algal.

[40] Martínková L, Stolz A, Rantwijk F, D'Antona N, Brady D, Otten LG, Riva S, Fessner W-D (2014). Nitrile converting enzymes involved in natural and synthetic cascade reactions. Cascade biocatalysis.

[41] Chhiba V, Bode M, Mathiba K, Brady D, Riva S, Fessner W-D (2014). Enzymatic stereoselective synthesis of β-amino acids. Cascade biocatalysis.

[42] Bigey F, Janbon G, Arnaud A, Galzy P (1995). Sizing of the *Rhodococcus* sp. R312 genome by pulsed-field gel electrophoresis. Localization of genes involved in nitrile degradation. Antonie Van Leeuwenhoek.

[43] Stoddard SF, Schmidt TM, Hein R, Roller BRK, Smith BJ (2014). rrnDB: improved tools for interpreting rRNA gene abundance in bacteria and archaea and a new foundation for future development. Nuc Acids Res.

[44] Zhang Z, Schwartz S, Wagner L, Miller W (2000). A greedy algorithm for aligning DNA sequences. J Comput Biol.

[45] McWilliam H, Li W, Uludag M, Squizzato S, Park YM, Buso N, Cowley AP, Lopez R (2013). Analysis tool web services from the EMBL-EBI. Nuc Acids Res.

[46] Li W, Cowley A, Uludag M, Gur T, McWilliam H, Squizzato S, Park YM, Buso N, Lopez R (2015). The EMBL-EBI bioinformatics web and programmatic tools framework. Nuc Acids Res.

[47] Sievers Fabian, Wilm Andreas, Dineen David, Gibson Toby J, Karplus Kevin, Li Weizhong, Lopez Rodrigo, McWilliam Hamish, Remmert Michael, Söding Johannes, Thompson Julie D, Higgins Desmond G (2011). Fast, scalable generation of high‐quality protein multiple sequence alignments using Clustal Omega. Molecular Systems Biology.

[48] Meier-Kolthoff JP, Auch AF, Klenk H-P, Göker M (2013). Genome sequence-based species delimitation with confidence intervals and improved distance functions. BMC Bioinform.

[49] Meier-Kolthoff JP, Hahnke RL, Petersen J, Scheuner C, Michael V, Fiebig A, Rohde C, Rohde M, Fartmann B, Goodwin LA (2014). Complete genome sequence of DSM 30083^T^, the type strain (U5/41^T^) of *Escherichia coli*, and a proposal for delineating subspecies in microbial taxonomy. Stand Genomic Sci.

[50] Meier-Kolthoff JP, Klenk H-P, Göker M (2014). Taxonomic use of DNA G+C content and DNA–DNA hybridization in the genomic age. Int J Syst Evol Microbiol.

[51] Van Domselaar GH, Stothard P, Shrivastava S, Cruz JA, Guo A, Dong X, Lu P, Szafron D, Greiner R, Wishart DS (2005). BASys: a web server for automated bacterial genome annotation. Nucl Acids Res.

[52] Tatusov RL, Galperin MY, Natale DA, Koonin EV (2000). The COG database: a tool for genome-scale analysis of protein functions and evolution. Nuc Acids Res.

[53] Tatusov RL, Koonin EV, Lipman DJ (1997). A genomic perspective on protein families. Science.

[54] Galperin MY, Makarova KS, Wolf YI, Koonin EV (2015). Expanded microbial genome coverage and improved protein family annotation in the COG database. Nuc Acids Res.

[55] Lowe TM, Eddy SR (1997). tRNAscan-SE: a program for improved detection of transfer RNA genes in genomic sequence. Nuc Acids Res.

[56] Chan PP, Lin B, Lowe TM (2019). tRNAscan-SE 2.0.

[57] Gardy JL, Brinkman FSL (2006). Methods for predicting bacterial protein subcellular localization. Nat Rev Microbiol.

[58] Pao SS, Paulsen IT, Saier MH (1998). Major facilitator superfamily. Microbiol Mol Biol Rev.

[59] Walmsley AR, Barrett MP, Bringaud F, Gould GW (1998). Sugar transporters from bacteria, parasites and mammals: structure–activity relationships. Trends Biochem Sci.

[60] Bassegoda A, Pastor FIJ, Diaz P (2012). *Rhodococcus* sp. strain CR-53 LipR, the first member of a new bacterial lipase family (family X) displaying an unusual Y-type oxyanion hole, similar to the *Candida antarctica* lipase clan. Appl Environ Microbiol.

[61] Zhang Y, Pan J, Luan Z-J, Xu G-C, Park S, Xu J-H (2014). Cloning and characterization of a novel esterase from *Rhodococcus* sp. for highly enantioselective synthesis of a chiral cilastatin precursor. Appl Environ Microbiol.

[62] Jiménez José I., Miñambres Baltasar, García José Luis, Díaz Eduardo (2004). Genomic Insights in the Metabolism of Aromatic Compounds in Pseudomonas. Pseudomonas.

[63] Yamada H, Kobayashi M (1996). Nitrile hydratase and its application to industrial production of acrylamide. Biosci Biotechnol Biochem.

[64] Nagasawa T, Takeuchi K, Yamada H (1988). Occurrence of a cobalt-induced and cobalt-containing nitrile hydratase in *Rhodococcus rhodochrous* J1. Biochem Biophys Res Commun.

[65] Thomas SM, DiCosimo R, Nagarajan V (2002). Biocatalysis: applications and potentials for the chemical industry. Trends Biotechnol.

[66] Ziemert N, Alanjary M, Weber T (2016). The evolution of genome mining in microbes – a review. Nat Prod Rep.

[67] Crits-Christoph A, Diamond S, Butterfield CN, Thomas BC, Banfield JF (2018). Novel soil bacteria possess diverse genes for secondary metabolite biosynthesis. Nature.

[68] Medema MH, Blin K, Cimermancic P, de Jager V, Zakrzewski P, Fischbach MA, Weber T, Takano E, Breitling R (2011). antiSMASH: rapid identification, annotation and analysis of secondary metabolite biosynthesis gene clusters in bacterial and fungal genome sequences. Nuc Acids Res.

[69] Blin K, Wolf T, Chevrette MG, Lu X, Schwalen CJ, Kautsar SA, Suarez Duran HG, de los Santos Emmanuel LC, Kim HU, Nave M (2017). antiSMASH 4.0—improvements in chemistry prediction and gene cluster boundary identification. Nuc Acids Res.

[70] Yu Q, Schaub P, Ghisla S, Al-Babili S, Krieger-Liszkay A, Beyer P (2010). The lycopene cyclase CrtY from *Pantoea ananatis* (formerly *Erwinia uredovora*) catalyzes an FAD_red_-dependent non-redox reaction. J Biol Chem.

[71] Oldfield E, Lin F-Y (2012). Terpene biosynthesis: modularity rules. Angew Chem Int Ed.

[72] Couvin D, Bernheim A, Toffano-Nioche C, Touchon M, Michalik J, Néron B, Rocha EPC, Vergnaud G, Gautheret D, Pourcel C (2018). CRISPRCasFinder, an update of CRISRFinder, includes a portable version, enhanced performance and integrates search for Cas proteins. Nuc Acids Res.

[73] Bertelli C, Laird MR, Williams KP, Lau BY, Hoad G, Winsor GL, Brinkman FS, Group SFURC (2017). IslandViewer 4: expanded prediction of genomic islands for larger-scale datasets. Nuc Acids Res.

[74] Szabó M, Kiss J, Olasz F (2010). Functional organization of the inverted repeats of IS30. J Bacteriol.

[75] Venter JC, Remington K, Heidelberg JF, Halpern AL, Rusch D, Eisen JA, Wu D, Paulsen I, Nelson KE, Nelson W (2004). Environmental genome shotgun sequencing of the Sargasso sea. Science.

[76] Hughes Martiny JB, Field D (2005). Ecological perspectives on the sequenced genome collection. Ecol Lett.

[77] Warren A, Archuleta J, Feng W-c, Setubal J (2010). Missing genes in the annotation of prokaryotic genomes. BMC Bioinf.

[78] Galperin MY, Koonin EV (2004). ‘Conserved hypothetical’ proteins: prioritization of targets for experimental study. Nuc Acids Res.

[79] Roberts RJ (2004). Identifying protein function - a call for community action. PLoS Biol.

[80] Frishman D (2007). Protein annotation at genomic scale: the current status. Chem Rev.

[81] Mills CL, Beuning PJ, Ondrechen MJ (2015). Biochemical functional predictions for protein structures of unknown or uncertain function. Comput Struct Biotechnol J.

[82] Delcher AL, Harmon D, Kasif S, White O, Salzberg SL (1999). Improved microbial gene identification with GLIMMER. Nuc Acids Res.

[83] Benson DA, Cavanaugh M, Clark K, Karsch-Mizrachi I, Lipman DJ, Ostell J, Sayers EW (2013). GenBank. Nuc Acids Res.

[84] Harrison PW, Lower RPJ, Kim NKD, Young JPW (2010). Introducing the bacterial ‘chromid’: not a chromosome, not a plasmid. Trends Microbiol.

[85] Jiménez JI, Miñambres B, García JL, Díaz E (2002). Genomic analysis of the aromatic catabolic pathways from *Pseudomonas putida* KT2440. Environ Microbiol.

[86] Zampolli J, Zeaiter Z, Di Canito A, Di Gennaro P (2019). Genome analysis and -omics approaches provide new insights into the biodegradation potential of *Rhodococcus*. Appl Microbiol Biotechnol.

[87] Riebel A, de Gonzalo G, Fraaije MW (2013). Expanding the biocatalytic toolbox of flavoprotein monooxygenases from *Rhodococcus jostii* RHA1. J Mol Catal B Enzym.

[88] Summers BD, Omar M, Ronson TO, Cartwright J, Lloyd M, Grogan G (2015). *E. coli* cells expressing the Baeyer–Villiger monooxygenase ‘MO14’ (*ro*03437) from *Rhodococcus jostii* RHA1 catalyse the gram-scale resolution of a bicyclic ketone in a fermentor. Org Biomol Chem.

[89] Van der Werf MJ (2000). Purification and characterization of a Baeyer–Villiger mono-oxygenase from *Rhodococcus erythropolis* DCL14 involved in three different monocyclic monoterpene degradation pathways. Biochem J.

[90] Rosłoniec KZ, Wilbrink MH, Capyk JK, Mohn WW, Ostendorf M, Van Der Geize R, Dijkhuizen L, Eltis LD (2009). Cytochrome P450 125 (CYP125) catalyses C26-hydroxylation to initiate sterol side-chain degradation in *Rhodococcus jostii* RHA1. Mol Microbiol.

[91] Grogan G (2011). Cytochromes P450: exploiting diversity and enabling application as biocatalysts. Curr Opin Chem Biol.

[92] Xiong F, Shuai J-J, Peng R-H, Tian Y-S, Zhao W, Yao Q-H, Xiong A-S (2010). Expression, purification and functional characterization of a recombinant 2,3-dihydroxybiphenyl-1,2-dioxygenase from *Rhodococcus rhodochrous*. Mol Biol Rep.

[93] Kuyukina MS, Ivshina IB, Serebrennikova MK, Krivoruchko AV, Korshunova IO, Peshkur TA, Cunningham CJ (2017). Oilfield wastewater biotreatment in a fluidized-bed bioreactor using co-immobilized *Rhodococcus* cultures. J Environ Chem Eng.

[94] Li S, Chaulagain MR, Knauff AR, Podust LM, Montgomery J, Sherman DH (2009). Selective oxidation of carbolide C–H bonds by an engineered macrolide P450 mono-oxygenase. Proc Natl Acad Sci U S A.

[95] Bugg TDH, Ahmad M, Hardiman EM, Singh R (2011). The emerging role for bacteria in lignin degradation and bio-product formation. Curr Opin Biotechnol.

[96] Whyte LG, Smits THM, Labbé D, Witholt B, Greer CW, van Beilen JB (2002). Gene cloning and characterization of multiple alkane hydroxylase systems in *Rhodococcus* strains Q15 and NRRL B-16531. Appl Environ Microbiol.

[97] Leipold F, Rudroff F, Mihovilovic MD, Bornscheuer UT (2013). The steroid monooxygenase from *Rhodococcus rhodochrous*; a versatile biocatalyst. Tetrahedron: Asymm.

[98] Toda H, Ohuchi T, Imae R, Itoh N (2015). Microbial production of aliphatic (*S*)-epoxyalkanes by using *Rhodococcus* sp. strain ST-10 styrene monooxygenase expressed in organic-solvent-tolerant *Kocuria rhizophila* DC2201. Appl Environ Microbiol.

[99] Vignali E, Tonin F, Pollegioni L, Rosini E (2018). Characterization and use of a bacterial lignin peroxidase with an improved manganese-oxidative activity. Appl Microbiol Biotechnol.

[100] van Beilen JB, Smits THM, Whyte LG, Schorcht S, Röthlisberger M, Plaggemeier T, Engesser K-H, Witholt B (2002). Alkane hydroxylase homologues in gram-positive strains. Environ Microbiol.

[101] Banerjee Amit (2000). Stereoselective Microbial Baeyer-Villiger Oxidations. Stereoselective Biocatalysis.

[102] Kostichka K, Thomas SM, Gibson KJ, Nagarajan V, Cheng Q (2001). Cloning and characterization of a gene cluster for cyclododecanone oxidation in *Rhodococcus ruber* SC1. J Bacteriol.

[103] Schumacher DJ, Fakoussa MR (1999). Degradation of alicyclic molecules by *Rhodococcus ruber* CD4. Appl Microbiol Biotechnol.

[104] Fink MJ, Rudroff F, Mihovilovic MD (2011). Baeyer–Villiger monooxygenases in aroma compound synthesis. Bioorg Med Chem Lett.

[105] Galinski EA, Pfeiffer H-P, Trüper HG (1985). 1,4,5,6-Tetrahydro-2-methyl-4-pyrimidinecarboxylic acid. A novel cyclic amino acid from halophilic phototrophic bacteria of the genus *Ectothiorhodospira*. Eur J Biochem.

[106] Roberts MF (2005). Organic compatible solutes of halotolerant and halophilic microorganisms. Saline Syst.

[107] García-Estepa R, Argandoña M, Reina-Bueno M, Capote N, Iglesias-Guerra F, Nieto JJ, Vargas C (2006). The *ectD* gene, which is involved in the synthesis of the compatible solute hydroxyectoine, is essential for thermoprotection of the halophilic bacterium *Chromohalobacter salexigens*. J Bacteriol.

[108] Pastor JM, Salvador M, Argandoña M, Bernal V, Reina-Bueno M, Csonka LN, Iborra JL, Vargas C, Nieto JJ, Cánovas M (2010). Ectoines in cell stress protection: uses and biotechnological production. Biotechnol Adv.

[109] Frings E, Sauer T, Galinski EA (1995). Production of hydroxyectoine: high cell-density cultivation and osmotic downshock of *Marinococcus* strain M52. J Biotechnol.

[110] Schiraldi C, Maresca C, Catapano A, Galinski EA, De Rosa M (2006). High-yield cultivation of *Marinococcus* M52 for production and recovery of hydroxyectoine. Res Microbiol.

[111] Tetali SD (2019). Terpenes and isoprenoids: a wealth of compounds for global use. Planta.

[112] Yamada Y, Kuzuyama T, Komatsu M, Shin-ya K, Omura S, Cane DE, Ikeda H (2015). Terpene synthases are widely distributed in bacteria. Proc Natl Acad Sci U S A.

[113] Dickschat JS (2016). Bacterial terpene cyclases. Nat Prod Rep.

[114] Bosello M, Robbel L, Linne U, Xie X, Marahiel MA (2011). Biosynthesis of the siderophore rhodochelin requires the coordinated expression of three independent gene clusters in *Rhodococcus jostii* RHA1. J Am Chem Soc.

[115] Wang H, Fewer DP, Holm L, Rouhiainen L, Sivonen K (2014). Atlas of nonribosomal peptide and polyketide biosynthetic pathways reveals common occurrence of nonmodular enzymes. Proc Natl Acad Sci U S A.

[116] Brandão PFB, Bull AT (2003). Nitrile hydrolysing activities of deep-sea and terrestrial mycolate actinomycetes. Antonie Van Leeuwenhoek.

[117] Brady D, Dube N, Petersen R (2006). Green chemistry: highly selective biocatalytic hydrolysis of nitrile compounds. S Afr J Sci.

[118] Velankar H, Clarke KG, Preez R, Cowan DA, Burton SG (2010). Developments in nitrile and amide biotransformation processes. Trends Biotechnol.

[119] O'Reilly C, Turner PD (2003). The nitrilase family of CN hydrolysing enzymes - a comparative study. J Appl Microbiol.

[120] Novikov AD, Lavrov KV, Kasianov AS, Gerasimova TV, Yanenko AS (2018). Draft genome sequence of *Rhodococcus* sp. strain M8, which can degrade a broad range of nitriles. Genome Announc.

[121] Yamaguchi T, Asano Y (2016). Draft genome sequence of an aldoxime degrader, *Rhodococcus* sp. strain YH3-3. Genome Announc.

[122] Gürtler V, Mayall BC, Seviour R (2004). Can whole genome analysis refine the taxonomy of the genus *Rhodococcus*?. FEMS Microbiol Rev.

[123] Okamoto S, Eltis LD (2007). Purification and characterization of a novel nitrile hydratase from *Rhodococcus* sp. RHA1. Mol Microbiol.

[124] Coffey L, Owens E, Tambling K, O’Neill D, O’Connor L, O’Reilly C (2010). Real-time PCR detection of Fe-type nitrile hydratase genes from environmental isolates suggests horizontal gene transfer between multiple genera. Antonie Van Leeuwenhoek.

[125] D'Antona N, Nicolosi G, Morrone R, Kubác D, Kaplan O, Martínková L (2010). Synthesis of novel cyano-cyclitols and their stereoselective biotransformation catalyzed by *Rhodococcus erythropolis* A4. Tetrahedron Asymmetry.

[126] Precigou S, Goulas P, Duran R (2001). Rapid and specific identification of nitrile hydratase (NHase)-encoding genes in soil samples by polymerase chain reaction. FEMS Microbiol Lett.

[127] Brandão PFB, Clapp JP, Bull AT (2003). Diversity of nitrile hydratase and amidase enzyme genes in *Rhodococcus erythropolis* recovered from geographically distinct habitats. Appl Environ Microbiol.

[128] Kobayashi M, Nishiyama M, Nagasawa T, Horinouchi S, Beppu T, Yamada H (1991). Cloning, nucleotide sequence and expression in *Escherichia coli* of two cobalt-containing nitrile hydratase genes from *Rhodococcus rhodochrous* J1. Biochim Biophys Acta Gene Struct Expr.

[129] Shen Y, Wang M, Li X, Zhang J, Sun H, Luo J (2012). Highly efficient synthesis of 5-cyanovaleramide by *Rhodococcus ruber* CGMCC3090 resting cells. J Chem Technol Biotechnol.

[130] Kubáč D, Čejková A, Masák J, Jirků V, Lemaire M, Gallienne E, Bolte J, Stloukal R, Martínková L (2006). Biotransformation of nitriles by *Rhodococcus equi* A4 immobilized in LentiKats®. J Mol Catal B Enzym.

[131] Vejvoda V, Šveda O, Kaplan O, Přikrylová V, Elišáková V, Himl M, Kubáč D, Pelantová H, Kuzma M, Křen V (2007). Biotransformation of heterocyclic dinitriles by *Rhodococcus erythropolis* and fungal nitrilases. Biotechnol Lett.

[132] Kobayashi M, Komeda H, Yanaka N, Nagasawa T, Yamada H (1992). Nitrilase from *Rhodococcus rhodochrous* J1. Sequencing and overexpression of the gene and identification of an essential cysteine residue. J Biol Chem.

[133] Luo H, Fan L, Chang Y, Ma J, Yu H, Shen Z (2010). Gene cloning, overexpression, and characterization of the nitrilase from *Rhodococcus rhodochrous* tg1-A6 in *E. coli*. Appl Biochem Biotechnol.

[134] Yeom S-J, Kim H-J, Lee J-K, Kim D-E, Oh D-K (2008). An amino acid at position 142 in nitrilase from *Rhodococcus rhodochrous* ATCC 33278 determines the substrate specificity for aliphatic and aromatic nitriles. Biochem J.

[135] Thuku RN, Brady D, Benedik MJ, Sewell BT (2009). Microbial nitrilases: versatile, spiral forming, industrial enzymes. J Appl Microbiol.

[136] Cobzaru C, Ganas P, Mihasan M, Schleberger P, Brandsch R (2011). Homologous gene clusters of nicotine catabolism, including a new ω-amidase for α-ketoglutaramate, in species of three genera of gram-positive bacteria. Res Microbiol.

[137] Webster NA, Ramsden DK, Hughes J. Comparative characterisation of two *Rhodococcus* species as potential biocatalysts for ammonium acrylate production. Biotechnol Lett. 2001;23(2):95–101.

[138] Kamal A, Kumar MS, Kumar CG, Shaik TB (2011). Bioconversion of acrylonitrile to acrylic acid by *Rhodococcus ruber* strain AKSH-84. J Microbiol Biotechnol.

[139] Coffey L, Clarke A, Duggan P, Tambling K, Horgan S, Dowling D, O’Reilly C (2009). Isolation of identical nitrilase genes from multiple bacterial strains and real-time PCR detection of the genes from soils provides evidence of horizontal gene transfer. Arch Microbiol.

[140] Kobayashi M, Nagasawa T, Yamada H (1989). Nitrilase of *Rhodococcus rhodochrous* J1. Purification and characterization. Eur J Biochem.

[141] Kobayashi M, Yanaka N, Nagasawa T, Yamada H (1990). Purification and characterization of a novel nitrilase of *Rhodococcus rhodochrous* K22 that acts on aliphatic nitriles. J Bacteriol.

[142] Pathak A, Chauhan A, Blom J, Indest KJ, Jung CM, Stothard P, Bera G, Green SJ, Ogram A (2016). Comparative genomics and metabolic analysis reveals peculiar characteristics of *Rhodococcus opacus* strain M213 particularly for naphthalene degradation. PLoS One.

[143] Grissa I, Pourcel C, Vergnaud G (2007). CRISPRFinder: a web tool to identify clustered regularly interspaced short palindromic repeats. Nuc Acids Res.

[144] Zhao H, Tian K, Qiu Q, Wang Y, Zhang H, Ma S, Jin S, Huo H (2018). Genome analysis of *Rhodococcus* sp. DSSKP-R-001: a highly effective β-estradiol-degrading bacterium. Int J Genomics.

[145] Di Gennaro P, Rescalli E, Galli E, Sello G, Bestetti G (2001). Characterization of *Rhodococcus opacus* R7, a strain able to degrade naphthalene and o-xylene isolated from a polycyclic aromatic hydrocarbon-contaminated soil. Res Microbiol.

[146] Arndt D, Grant JR, Marcu A, Sajed T, Pon A, Liang Y, Wishart DS (2016). PHASTER: a better, faster version of the PHAST phage search tool. Nuc Acids Res.

[147] Zheng G-W, Xu J-H (2011). New opportunities for biocatalysis: driving the synthesis of chiral chemicals. Curr Opin Biotechnol.

[148] Panke S, Wubbolts M (2005). Advances in biocatalytic synthesis of pharmaceutical intermediates. Curr Opin Chem Biol.

[149] Jemli S, Ayadi-Zouari D, Hlima HB, Bejar S (2016). Biocatalysts: application and engineering for industrial purposes. Crit Rev Biotechnol.

[150] Pollard DJ, Woodley JM (2007). Biocatalysis for pharmaceutical intermediates: the future is now. Trends Biotechnol.

[151] Yam KC, Okamoto S, Roberts JN, Eltis LD (2011). Adventures in *Rhodococcus* - from steroids to explosives. Can J Microbiol.

[152] Koren S, Walenz BP, Berlin K, Miller JR, Bergman NH, Phillippy AM (2017). Canu: scalable and accurate long-read assembly via adaptive *k*-mer weighting and repeat separation. Genome Res.

[153] Li H, Durbin R (2010). Fast and accurate long-read alignment with burrows–wheeler transform. Bioinformatics.

[154] Walker BJ, Abeel T, Shea T, Priest M, Abouelliel A, Sakthikumar S, Cuomo CA, Zeng Q, Wortman J, Young SK (2014). Pilon: an integrated tool for comprehensive microbial variant detection and genome assembly improvement. PLoS One.

[155] Aziz R, Bartels D, Best A, DeJongh M, Disz T, Edwards R, Formsma K, Gerdes S, Glass E, Kubal M (2008). The RAST server: rapid annotations using subsystems technology. BMC Genomics.

[156] Brettin T, Davis JJ, Disz T, Edwards RA, Gerdes S, Olsen GJ, Olson R, Overbeek R, Parrello B, Pusch GD (2015). RASTtk: a modular and extensible implementation of the RAST algorithm for building custom annotation pipelines and annotating batches of genomes. Sci Rep.

[157] Overbeek R, Olson R, Pusch GD, Olsen GJ, Davis JJ, Disz T, Edwards RA, Gerdes S, Parrello B, Shukla M (2014). The SEED and the rapid annotation of microbial genomes using subsystems technology (RAST). Nuc Acids Res.

