# Supplementary material for: The complete genome sequence of the nitrile biocatalyst Rhodocccus rhodochrous ATCC BAA-870
Source: BMC Genomics. 2020 Jan 2;21:3. doi: 10.1186/s12864-019-6405-7 (PMC6941271; doi:10.1186/s12864-019-6405-7)
Supplement: Supplementary file 1 — Additional file 1: SuppInfo Frederick et al. BAA-870 genome. Table S1. All sequenced Rhodococcus strains (353) according to the NCBI database (accessed 13/03/2019). Table S2. All complete sequenced Rhodococcus species ranked by release date according to the NCBI Genome database (accessed 11/03/2019). Table S3. Whole genome distance statistics between Rhodococcus rhodochrous ATCC BAA-870 and two closely matched strains. Table S4. Rhodococcus rhodochrous ATCC BAA-870 protein function breakdown based on BASys annotation COG classifications. [file 12864_2019_6405_MOESM1_ESM.pdf]

Supplementary Information belonging to:

The complete genome sequence of the nitrile biocatalyst *Rhodocccus rhodochrous* ATCC BAA-870

Joni Frederick, Fritha Hennessy, Uli Horn, Pilar de la Torre Cortés, Marcel van den Broek, Ulrich Strych, Richard Willson, Charles A. Hefer, Jean-Marc G. Daran, Trevor Sewell, Linda G. Otten[§], Dean Brady[§]

[§]Corresponding authors: dbrady@wits.ac.za; l.g.otten@tudelft.nl

Table of Contents

Table S1. All sequenced *Rhodococcus* strains (353) according to the NCBI database (accessed 13/03/2019)

Table S2. All complete sequenced *Rhodococcus* species ranked by release date according to the NCBI Genome database (accessed 11/03/2019)

Table S3. Whole genome distance statistics between *Rhodococcus rhodochrous* ATCC BAA-870 and two closely matched strains

Table S4. *Rhodococcus rhodochrous* ATCC BAA-870 protein function breakdown based on BASys annotation COG classifications

References

Table S1. All sequenced *Rhodococcus* strains (353) according to the NCBI Genome database (accessed 13/03/2019).

Organism	# Genome assemblies	Medium total length (Mbp)	Plasmids	Median protein count	proteins /Mbp	Median G + C %
Rhodococcus (uncharacterized isolates)	158	5.9	26 (in 13 strains)	5314	899	65
R. hoagii (previously known as *R. equi*)	40	5.2	7 (in 5 strains)	4725	908	69
R. fascians	29	5.9	2 [1]	5354	907	64
R. erythopolis	26	6.9	12 (in 7 strains)	6233	907	62
R. rhodochrous	13	6.1	NC_008823 [2]	5279	865	68
R. opacus	11	8.8	21 (in 4 strains)	7837	887	67
R. ruber	11	5.6	4 (in 2 strain)	4878	874	71
R. pyridinivorans	10	5.3	2 (in 1 strain)	4644	879	68
R. qingshengii	8	6.7	3 (in 1 strain)	5999	895	62
R. wratislaviensis	5	9.5	0	8187	858	67
R. koreensis	3	10.1	0	8817	872	67
R. jostii	3	9.7	3 (in 1 strain)	8473	871	67
R. triatomae	3	4.7	0	4297	907	69
R. rhodnii	3	4.5	0	4036	905	70
R. enclensis	2 (WGS)	7.5	0	6826	913	62
R. globerulus	2 (WGS)	6.8	0	6053	896	62
R. aetherivorans	2	6.1	3 (in 2 strains)	5257	865	70
R. phenolicus	2	5.6	0	5600	998	68
R. kyotonensis	2 (WGS)	5.9	0	5295	899	65
R. maanshanensis	2 (WGS)	5.7	0	4973	877	69
R. tukisamuensis	2	5.5	0	4769	869	70

R. gordoniae	2 (WGS)	4.9	0	4262	879	68
R. coprophilus	2	4.6	0	4080	894	67
R. corynebacterioides	2	3.9	0	3504	890	70
R. imtechensis RKJ300 = JCM 13270	1 ([3] and WGS)	8.2	0	7245	881	67
R. baikonurensis JCM 18801	1 (WGS)	6.8	nd	nd	-	62
R. yunnanensis NBRC 103083	1 (WGS)	6.4	0	5650	886	64
R. kunmingensis DSM 45001	1 (WGS)	5.6	0	4885	869	66
R. agglutinans CCTCC AB2014297	1	5.4	0	4853	895	69
R. zopfii NBRC 100606 = JCM 9919	1 (2x WGS)	5.3	0	5575	1060	67
R defluvii Ca11	1 (WGS)	5.1	0	4535	883	69
R. biphenylivorans TG9	1	5.0	0	4373	869	68
R. marinonascens NBRC 14363	1 (WGS)	4.9	0	4269	867	64
R. kroppenstedtii DSM 44908	1 (2x WGS)	4.0	0	3554	889	70
**Total / Average**	343	6.1 ± 1.6		5467 ± 1391	894 ± 39	67 ± 3

Strains that were sequenced more than once were only counted 1 time. WGS = whole genome shotgun sequence, no reference available; nd = not determined.

Table S2: All complete sequenced *Rhodococcus* species ranked by species according to the NCBI Genome database (accessed 11/03/2019).

Organism/ Name	BioProject	Assembly	Size (Mb)	GC%	Replicons	Pro-tein	Release Date
Rhodococcus aetherivorans BCP1 [1]	PRJNA213668	GCA_000470885.1	6.2	70.29	**chromosome**:NZ_CM002177.1/CM002177.1<br>**plasmid** pBMC1:NZ_CM002178.1/CM002178.1<br>**plasmid** pBMC2:NZ_CM002179.1/CM002179.1<br>**plasmid** pBMC2:NZ_CM002179.1/CM002179.1	5495	2013/09/24
Rhodococcus aetherivorans IcdP1	PRJNA281168	GCA_000982715.1	5.9	70.60	**chromosome**:NZ_CP011341.1/CP011341.1	5020	2015/04/30
Rhodococcus biphenylivorans TG9	PRJNA390866	GCA_003288095.1	5.0	68.0	**chromosome**: NZ_CP022208.1/CP022208.1	4373	2018/07/05
Rhodococcus coprophilus NCTC10994	PRJEB6403	GCA_900478115.1	4.6	66.80	**chromosome** 1:NZ_LS483468.1/LS483468.1	4081	2018/06/17
Rhodococcus erythropolis BG43	PRJNA280916	GCA_000975175.1	6.9	62.28	**chromosome**:NZ_CP011295.1/CP011295.1<br>**plasmid** pRLCBG43:NZ_CP011296.1/CP011296.1<br>**plasmid** pRLLBG43:NZ_CP011297.1/CP011297.1<br>**plasmid** pRLLBG43:NZ_CP011297.1/CP011297.1	6233	2015/04/21
Rhodococcus erythropolis CCM2595	PRJNA81583	GCA_000454045.1	6.4	62.50	**chromosome**:NC_022115.1/CP003761.1<br>**plasmid** pRECF1:NC_022125.1/CP003762.1	5776	2013/08/22
Rhodococcus erythropolis PR4 (= NBRC 100887)	PRJDA20395	GCA_000010105.1	6.9	62.29	**chromosome**:NC_012490.1/AP008957.1<br>**plasmid** pREC1:NC_007486.1/AP008932.1<br>**plasmid** pREC2:NC_007487.1/AP008933.1<br>**plasmid** pREL1:NC_007491.1/AP008931.1	6321	2009/04/01
Rhodococcus erythropolis R138	PRJNA188397	GCA_000696675.2	6.8	62.33	**chromosome**:NZ_CP007255.1/CP007255.1<br>**plasmid** pCRE138:NZ_CP007256.1/CP007256.1<br>**plasmid** pLRE138:NZ_CP007257.1/CP007257.1	6130	2015/12/30

Rhodococcus fascians D188	PRJNA313428	GCA_001620305.1	5.5	64.58	**chromosome**:NZ_CP015235.1/CP015235.1<br>**plasmid** pFiD188:NZ_CP015236.1/CP015236.1<br>**plasmid** unnamed2:NZ_CP015237.1/CP015237.1	5015	2016/04/14
Rhodococcus hoagii 103S	PRJEA41335	GCA_000196695.1	5.0	68.80	**chromosome**:NC_014659.1/FN563149.1	4540	2010/11/09
Rhodococcus hoagii ATCC 33707 [2]	PRJNA31525	GCA_000164155.2	5.3	68.70	**chromosome**:NZ_CM001149.1/CM001149.1	4775	2010/05/05
Rhodococcus hoagii DSSKP-R-001	PRJNA438171	GCA_003013675.1	5.4	68.66	**chromosome**:NZ_CP027793.1/CP027793.1<br>**plasmid** plas1:NZ_CP027794.1/CP027794.1<br>**plasmid** plas2:NZ_CP027795.1/CP027795.1	4987	2018/03/22
Rhodococcus jostii RHA1	PRJNA13693	GCA_000014565.1	9.7	66.97	**chromosome**:NC_008268.1/CP000431.1<br>**plasmid** pRHL1:NC_008269.1/CP000432.1<br>**plasmid** pRHL2:NC_008270.1/CP000433.1<br>**plasmid** pRHL3:NC_008271.1/CP000434.1	8690	2006/07/28
Rhodococcus opacus 1CP	PRJNA253567	GCA_001685605.1	8.6	67.03	**chromosome**:NZ_CP009111.1/CP009111.1<br>**plasmid** pR1CP1:NZ_CP009112.1/CP009112.1<br>**plasmid** pR1CP2:NZ_CP009113.1/CP009113.1	7380	2016/07/15
Rhodococcus opacus B4	PRJDA34839	GCA_000010805.1	8.8	67.63	**chromosome**:NC_012522.1/AP011115.1<br>**plasmid** pKNR:NC_012523.1/AP011118.1<br>**plasmid** pKNR01:NC_006969.2/AP011119.1<br>**plasmid** pKNR02:NC_006970.2/AP011120.1<br>**plasmid** pROB01:NC_012520.1/AP011116.1<br>**plasmid** pROB02:NC_012521.1/AP011117.1	7837	2009/04/01
Rhodococcus opacus PD630	PRJNA178618	GCA_000599545.1	9.2	67.19	**chromosome**:NZ_CP003949.1/CP003949.1<br>**plasmid** 1:NZ_CP003950.1/CP003950.1<br>**plasmid** 2:NZ_CP003951.1/CP003951.1<br>**plasmid** 3:NZ_CP003952.1/CP003952.1<br>**plasmid** 4:NZ_CP003953.1/CP003953.1<br>**plasmid** 5:NZ_CP003954.1/CP003954.1<br>**plasmid** 6:NZ_CP003955.1/CP003955.1<br>**plasmid** 7:NZ_CP003956.1/CP003956.1	7903	2014/02/28

					**plasmid** 8:NZ_CP003957.1/CP003957.1<br>**plasmid** 9:NZ_CP003958.1/CP003958.1		
Rhodococcus opacus R7 [3]	PRJNA246296	GCA_000736435.1	10.1	66.88	**chromosome**:NZ_CP008947.1/CP008947.1<br>**plasmid** pPDG1:NZ_CP008948.1/CP008948.1<br>**plasmid** pPDG2:NZ_CP008949.1/CP008949.1<br>**plasmid** pPDG3:NZ_CP008950.1/CP008950.1<br>**plasmid** pPDG4:NZ_CP008951.1/CP008951.1<br>**plasmid** pPDG5:NZ_CP008952.1/CP008952.1	8731	2014/07/30
Rhodococcus pyridinivorans GF3	PRJNA398330	GCA_002269365.1	5.3	67.90	**chromosome**:NZ_CP022915.1/CP022915.1	4644	2017/08/24
Rhodococcus pyridinivorans SB3094	PRJNA231235	GCA_000511305.1	5.6	67.81	**chromosome**:NC_023150.1/CP006996.1<br>**plasmid** unnamed:NC_023144.1/CP006997.1<br>**plasmid** unnamed2:NC_023145.1/CP006998.1	4893	2013/12/20
Rhodococcus qingshengii djl-6-2	PRJNA429114	GCA_002893965.1	6.7	62.39	**chromosome** 1:NZ_CP025959.1/CP025959.1<br>**plasmid** pDJL1:NZ_CP025960.1/CP025960.1<br>**plasmid** pDJL2:NZ_CP025961.1/CP025961.1<br>**plasmid** pDJL3:NZ_CP025962.1/CP025962.1	6160	2018/01/22
Rhodococcus rhodochrous EP4 [4]	PRJNA437303	GCA_003004765.2	5.7	67.90	**chromosome**:NZ_CP032221.1/CP032221.1	4942	2018/09/18
Rhodococcus rhodochrous NCTC10210	PRJEB6403	GCA_900187265.1	5.3	68.20	**chromosome** 1:NZ_LT906450.1/LT906450.1	4668	2017/08/15
Rhodococcus ruber P14	PRJNA415879	GCA_002741725.1	5.5	70.50	**chromosome**:NZ_CP024315.1/CP024315.1	4869	2017/11/02
Rhodococcus ruber SD3	PRJNA450724	GCA_003086595.1	5.4	70.60	**chromosome**:NZ_CP029146.1/CP029146.1	4768	2018/05/03
Rhodococcus ruber YC-YT1	PRJNA401381	GCA_003586525.1	5.9	70.24	**chromosome**:NZ_CP023714.1/CP023714.1<br>**plasmid** unnamed1:NZ_CP023712.1/CP023712.1<br>**plasmid** unnamed2:NZ_CP023713.1/CP023713.1	5181	2018/09/21
Rhodococcus ruber YYL	PRJNA415353	GCA_002863905.1	5.9	70.29	**chromosome**:NZ_CP024890.1/CP024890.1<br>**plasmid** pYYL1.1:NZ_CP024892.1/CP024892.1<br>**plasmid** pYYL1.2:NZ_CP024891.1/CP024891.1	5266	2018/01/05

Rhodococcus sp. 008	PRJNA296603	GCA_001682295.1	7.4	62.35	**chromosome**:NZ_CP012749.1/CP012749.1<br>**plasmid** pR8C1:NZ_CP015204.1/CP015204.1<br>**plasmid** pR8C2:NZ_CP015205.1/CP015205.1<br>**plasmid** pR8L1:NZ_CP015203.1/CP015203.1	6767	2016/07/08
Rhodococcus sp. 2G	PRJNA353162	GCA_001886355.1	5.6	67.51	**chromosome**:NZ_CP018063.1/CP018063.1<br>**plasmid** p1:NZ_CP018064.1/CP018064.1	4930	2016/11/28
Rhodococcus sp. B7740	PRJNA264767	GCA_000954115.1	5.3	64.90	**chromosome**:NZ_CP010797.1/CP010797.1	4875	2015/03/17
Rhodococcus sp. BH4	PRJNA313101	GCA_002079265.1	7.0	62.34	**chromosome**:NZ_CP014941.1/CP014941.1<br>**plasmid** unnamed:NZ_CP014942.1/CP014942.1	6362	2017/04/05
Rhodococcus sp. H-CA8f	PRJNA411856	GCA_002501585.1	6.5	62.49	**chromosome**:NZ_CP023720.1/CP023720.1<br>**plasmid** unnamed:NZ_CP023721.1/CP023721.1	5979	2017/10/10
Rhodococcus sp. MTM3W5.2	PRJNA357717	GCA_001984015.1	5.7	69.00	**chromosome**:NZ_CP019572.1/CP019572.1	4272	2017/02/03
Rhodococcus sp. NJ-530	PRJNA506529	GCA_003860625.1	7.3	62.30	**chromosome**:NZ_CP034152.1/CP034152.1<br>**plasmid** unnamed1:NZ_CP034153.1/CP034153.1<br>**plasmid** unnamed2:NZ_CP034154.1/CP034154.1<br>**plasmid** unnamed3:NZ_CP034155.1/CP034155.1<br>**plasmid** unnamed4:NZ_CP034156.1/CP034156.1	6491	2018/12/04
Rhodococcus sp. P1Y	PRJNA494794	GCA_003641205.1	5.9	63.20	**chromosome**:NZ_CP032762.1/CP032762.1	5253	2018/10/15
Rhodococcus sp. p52	PRJNA255270	GCA_000763325.2	5.4	67.85	**chromosome**:NZ_CP016819.1/CP016819.1<br>**plasmid** pDF01:NZ_CP016821.1/CP016821.1<br>**plasmid** pDF02:NZ_CP016820.1/CP016820.1<br>**plasmid** pDF03:NZ_CP016822.1/CP016822.1	4753	2016/08/16
Rhodococcus sp. PBTS 1	PRJNA313428	GCA_001620025.1	4.3	70.00	**chromosome**:NZ_CP015219.1/CP015219.1	3793	2016/04/13
Rhodococcus sp. PBTS 2	PRJNA313428	GCA_001620005.1	5.3	64.70	**chromosome**:NZ_CP015220.1/CP015220.1<br>**plasmid** unnamed1:NZ_CP015221.1/CP015221.1	4864	2016/04/13

Rhodococcus sp. S2-17	PRJNA386772	GCA_003130705.1	8.0	65.59	**chromosome**:NZ_CP021354.1/CP021354.1<br>**plasmid** pRB11:NZ_CP021357.1/CP021357.1<br>**plasmid** pRB29:NZ_CP021356.1/CP021356.1<br>**plasmid** pRB98:NZ_CP021355.1/CP021355.1	6877	2018/05/17
Rhodococcus sp. WB1	PRJNA317416	GCA_001700945.1	6.1	70.48	**chromosome**:NZ_CP015529.1/CP015529.1<br>**plasmid** pWB1:NZ_CP015530.1/CP015530.1	5390	2016/08/09
Rhodococcus sp. WMMA185	PRJNA338664	GCA_001767395.1	4.4	64.10	**chromosome**:NZ_CP017014.1/CP017014.1	3885	2016/10/18
Rhodococcus sp. X156	PRJNA511906	GCA_004006015.1	3.7	72.20	**chromosome**:NZ_CP034766.1/CP034766.1	3389	2019/01/09
Rhodococcus sp. YL-1	PRJNA343344	GCA_001942025.1	7.6	62.37	**chromosome**:NZ_CP017299.1/CP017299.1<br>**plasmid** pYLC1:NZ_CP017300.1/CP017300.1<br>**plasmid** pYLC2:NZ_CP017301.1/CP017301.1<br>**plasmid** pYLC3:NZ_CP017302.1/CP017302.1<br>**plasmid** pYLL1:NZ_CP017303.1/CP017303.1<br>**plasmid** pYLL2:NZ_CP017304.1/CP017304.1	6887	2017/01/10

Notes:
[1] WGS AVAE01 - 3 scaffolds; [2] WGS ADNW02 - 1 scaffold; [3] 6 scaffolds; [4] 1 scaffold.

Table S3. Whole genome distance statistics between Rhodococcus rhodochrous ATCC BAA-870 and two closely matched strains

	**Closely matched strains based on 16S rRNA sequences**		**Comparative strain**
	R. pyridinovorans **SB3094**	***Rhodococcus*** **sp. 2G**	***R. jostii*** **RHA1**
**Formula: 1 (HSP length / total length)**			
Distance	0.1860	0.2189	0.7957
DDH estimate (GLM-based)	69.50% [65.6 - 73.2%]	63.60% [59.8 - 67.2%]	17.20% [14.2 - 20.7%]
Probability that DDH > 70% (i.e., same species)	73.1% (via logistic regression)	54.81% (via logistic regression)	0% (via logistic regression)
Probability that DDH > 79% (i.e., same subspecies)	28.94% (via logistic regression)	14.92% (via logistic regression)	0% (via logistic regression)
**Formula: 2 (identities / HSP length)**			
Distance	0.0486	0.0504	0.2241
DDH estimate (GLM-based)	61.80% [58.9 - 64.6%]	60.70% [57.9 - 63.5%]	19.60% [17.4 - 22%]
Probability that DDH > 70% (i.e., same species)	58.07% (via logistic regression)	54.7% (via logistic regression)	0% (via logistic regression)
Probability that DDH > 79% (i.e., same subspecies)	15.34% (via logistic regression)	13.91% (via logistic regression)	0% (via logistic regression)

**Formula: 3 (identities / total length)**			
Distance	0.2256	0.2583	0.8415
DDH estimate (GLM-based)	70.20% [66.7 - 73.4%]	64.60% [61.3 - 67.9%]	16.90% [14.3 - 19.9%]
Probability that DDH > 70% (i.e., same species)	74.54% (via logistic regression)	48.24% (via logistic regression)	0% (via logistic regression)
Probability that DDH > 79% (i.e., same subspecies)	23.9% (via logistic regression)	12.1% (via logistic regression)	0% (via logistic regression)
**Difference in % G+C**			
	0.15 (interpretation: either distinct or same species)	0.01 (interpretation: either distinct or same species)	0.32 (interpretation: either distinct or same species)

Rhodococcus rhodochrous ATCC BAA-870 serves as the reference strain. *R. jostii* RHA1 has been added for comparison.

Table S4: ***Rhodococcus rhodochrous*** **ATCC BAA-870 protein function breakdown based on BASys annotation COG classifications**

	Chromosome gene count	Plasmid gene count	Total gene count
**BASys assigned Clusters of Orthologous Groups**			
C - Energy production and conversion	264	35	299
D - Cell division and chromosome partitioning	24	4	28
E - Amino acid transport and metabolism	257	14	271
F - Nucleotide transport and metabolism	65	2	67
G - Carbohydrate transport and metabolism	186	20	206
H - Coenzyme metabolism	112	9	121
I - Lipid metabolism	382	29	411
J - Translation, ribosomal structure and biogenesis	122	2	124
K - Transcription	293	29	322
L - DNA replication, recombination and repair	117	38	155
M - Cell envelope biogenesis, outer membrane	106	2	108
N - Secretion, motility and chemotaxis	1	0	1

O - Posttranslational modification, protein turnover, chaperones	97	2	99
P - Inorganic ion transport and metabolism	216	20	236
Q - Secondary metabolites biosynthesis, transport and catabolism	151	23	174
R - General function prediction only	360	31	391
S - COG of unknown function	173	4	177
T - Signal transduction mechanisms	111	7	118
A,B,U,Y,Z - other COG categories[1]	69	7	76
Total genes assigned a COG	3038	271	3309
**Unknown genes**	3764	399	4163
**Total genes**	6871	677	7548

Gene counts are calculated based on the protein function fractions reported by BASys annotation of the chromosome and plasmid.

[1] not broken down in separate COG categories by BASys

References

1. Stamler RA, Vereecke D, Zhang Y, Schilkey F, Devitt N, Randall JJ. Complete genome and plasmid sequences for *Rhodococcus fascians* D188 and draft sequences for *Rhodococcus* isolates PBTS 1 and PBTS 2. 2016;4(3):e00495-00416.
2. Matsui T, Saeki H, Shinzato N, Matsuda H. Analysis of the 7.6-kb cryptic plasmid pNC500 from *Rhodococcus rhodochrous* B-276 and construction of *Rhodococcus–E. coli* shuttle vector. Appl Microbiol Biotechnol. 2007; 74(1): 169-175.
3. Vikram S, Kumar S, Subramanian S, Raghava GPS. Draft genome sequence of the nitrophenol-degrading actinomycete *Rhodococcus imtechensis* RKJ300. J Bacteriol. 2012;194(13):3543.
